# Vibrational Spectroscopy in Studies of Atmospheric Corrosion

**DOI:** 10.3390/ma10040413

**Published:** 2017-04-18

**Authors:** Saman Hosseinpour, Magnus Johnson

**Affiliations:** 1 Max Planck Institute for Polymer Research, Ackermannweg 10, 55128 Mainz, Germany; hosseinpour@mpip-mainz.mpg.de; 2 Department of Chemistry, Division of Surface and Corrosion Science, KTH Royal Institute of Technology, SE-100 44 Stockholm, Sweden

**Keywords:** atmospheric corrosion, infrared spectroscopy, Raman spectroscopy, vibrational sum frequency spectroscopy, VSFS, SFG

## Abstract

Vibrational spectroscopy has been successfully used for decades in studies of the atmospheric corrosion processes, mainly to identify the nature of corrosion products but also to quantify their amounts. In this review article, a summary of the main achievements is presented with focus on how the techniques infrared spectroscopy, Raman spectroscopy, and vibrational sum frequency spectroscopy can be used in the field. Several different studies have been discussed where these instruments have been used to assess both the nature of corrosion products as well as the properties of corrosion inhibitors. Some of these techniques offer the valuable possibility to perform in-situ measurements in real time on ongoing corrosion processes, which allows the kinetics of formation of corrosion products to be studied, and also minimizes the risk of changing the surface properties which may occur during ex-situ experiments. Since corrosion processes often occur heterogeneously over a surface, it is of great importance to obtain a deeper knowledge about atmospheric corrosion phenomena on the nano scale, and this review also discusses novel vibrational microscopy techniques allowing spectra to be acquired with a spatial resolution of 20 nm.

## 1. Introduction

The main aim of this review article is to illustrate how different vibrational spectroscopy techniques are used in the field of atmospheric corrosion. Since the focus of this article is on the ability of the various techniques, the main divisions of the sections have been based on experimental technique rather than the metal or alloy studied, which is presented in subsection.

A significant number of the studies discussed here are model studies of metal surfaces exposed to one or a few corrosive gases, thus in significantly less complex atmospheres than in natural environments. Hence, the number of corrosion products formed will be lower, allowing for simpler spectral assignments. Several of the studies discussed below focus on investigating indoor atmospheric corrosion processes, which significantly differ from corrosion occurring outdoors due to the different nature of the corrosive species present among other parameters. A summary of important gases found in indoor environments is found in Table 1.

Two important properties of some of the vibrational spectroscopy techniques (e.g., infrared reflection/absorption spectroscopy) are the ability to perform experiments in-situ and in real time, as well as obtaining chemical information with a spatial resolution of 20 nm with the novel technique nano FTIR (Fourier transform infrared spectroscopy) microscopy. These two abilities, are crucial parameters in revealing the mechanisms of atmospheric corrosion processes.

In several of the studies discussed below, complementary techniques such as scanning electron microscopy (SEM), X-ray photoelectron spectroscopy (XPS), quartz crystal microbalance (QCM), cathodic reduction (CR), and atomic force microscopy (AFM), have been used to characterize the corrosion products, with the obvious advantage that the corrosion process can be examined from different viewpoints. Although here the results obtained by these other techniques have not been discussed in detail, the complementary techniques employed have been mentioned to demonstrate the added value of combining various analytical techniques.

## 2. Results and Discussion

### 2.1. Overwiew

The vibrational spectroscopy techniques that are described below are infrared, Raman, and vibrational sum frequency spectroscopy, and for each of these main techniques different subtypes are described. Each section starts with a brief description of the theory behind the technique, and is followed by a discussion how the technique has been used to study atmospheric corrosion of different metals and alloys.

IR and Raman spectroscopy have different advantages and drawbacks when applied to studies of atmospheric corrosion, and before summarizing specific studies a brief comparison between the techniques is provided.

The spectral range covered by IR and Raman spectroscopy commonly differs, and usually Raman spectroscopy can offer a wider accessible range in the fingerprint and group frequency regions. In Raman spectroscopy the spectral region ~100–4000 cm^−1^ is commonly covered, whereas in IR spectroscopy the lowest reachable wavenumber is usually around 600 cm^−1^ due to the cutoff frequency of MCT detectors. With other IR detectors such as DTGS it is however possible to reach down to around 80 cm^−1^, thus similar to Raman spectroscopy. However, even with other detectors than MCT, the accessible wavenumber range with IR can be limited at lower wavenumbers by the transparency of the windows possibly used in the system.

In studies of local corrosion, confocal Raman microscopy has been used in a number of atmospheric corrosion studies, and provides a spatial resolution of some hundreds of nm, which is significantly better than conventional IR microscopy which at the best has a spatial resolution of around 5 μm due to diffraction limitation. However, with novel techniques such as nano FTIR microscopy it is possible to study the distribution of corrosion products with a spatial resolution of 20 nm.

Infrared reflection/absorption spectroscopy (IRRAS) has commonly been used to perform in-situ studies during an ongoing atmospheric corrosion process, and although such investigations would be possible with Raman spectroscopy as well, such studies are rare.

IR spectroscopy has the advantage that the measured absorbance is directly proportional to the amount of corrosion products formed, and hence quantitative measurements can be performed. Raman spectroscopy is more of a qualitative technique, since the surface topography (e.g., surface scratches and chunks of corrosion products) affect the scattered Raman signal.

Raman spectroscopy and some IR techniques such as IRRAS do not require any sample preparation, which however is required for IR transmission and IR attenuated total reflection (ATR) studies of corrosion products that have been scraped off the sample. In Raman spectroscopy the corroded surface can be either shiny or dull, whereas in IRRAS a shiny sample is desired in order to have as much IR radiation as possible reaching the detector. Further, IR spectroscopy requires the acquisition of a background spectrum from, e.g., a gold surface or an uncorroded surface (frequently used in in-situ IRRAS measurements).

A limiting parameter in performing Raman spectroscopy on corrosion products is the fluorescence that usually arises from corrosion products. For instance, colorful corrosion products on copper (Cu I or II oxide) cause a large fluorescence background, which sometimes overwhelms the vibrational signature of the corrosion products. However, in some cases such fluorescence generation has been used as an indication of the formation of specific corrosion products.

To conclude, the discussion above reveals that IR and Raman spectroscopy have different advantages in examinations of corrosion products, and thus depending on the specific system that is of interest, the most favorable technique should be chosen. In many situations these two spectroscopic techniques can provide complementary information and could thus be combined.

#### 2.1.1. Infrared Spectroscopy in General

Infrared (IR) spectroscopy owing to its abilities in providing chemical information is a widely used technique in numerous research fields, and has also found great use in the field of atmospheric corrosion, with the main aim of identifying the corrosion products formed. However, with certain input parameters IR spectroscopy can also be used to quantify the amount of corrosion products. IR spectroscopy has been described in a large amount of sources [3,4], and hence only a brief description is provided here.

In infrared spectroscopy molecular vibrations are probed, and since different functional groups absorb IR radiation of different wavelengths, the technique can be used to assist in the identification of unknown compounds. The selection rules for IR spectrocopy are that the dipole moment has to change during the vibration, and that the IR radiation must have a wavelength that corresponds to the energy difference between vibrational states. In infrared reflection/absorption spectroscopy (IRRAS) and additional selection rule for metal surfaces is that the molecular vibration must have a component that is perpendicular to the surface plane.

IR spectroscopy can be performed in various modes depending on the type of sample that is to be studied [5]. In the transmission mode, the corrosion products are scraped off from the surface, mixed with for example KBr, and pressed into a pellet. The transmitted IR radiation through the pellet is then measured. In order to use this technique, a fairly large amount of corrosion products is required, since it is necessary to scrape them off from the surface and mix them into the pellet. In the attenuated total reflection (ATR) mode (ATR), corrosion products also need to be scraped off from the sample, and they are placed on a crystal (e.g., diamond), which guides the IR beam to the sample in order to acquire a spectrum. An advantage of ATR in comparison to transmission IR is that the corrosion products can be studied directly, without the need of making a pellet. In diffuse reflectance (DRIFTS) the diffusively reflected light from the powder of corrosion products is detected. When using infrared reflection/absorption spectroscopy (IRRAS), an IR beam incident at a grazing angle of incidence (~80°) from the surface normal is reflected off the metal surface and detected. The advantage of IRRAS is that ultrathin layers of corrosion products can be examined in-situ, and hence the technique is well suited for studies of atmospheric corrosion processes. It also has the ability to provide information about thin films of corrosion inhibitors. An advantage of IRRAS is that no sample preparation is required (e.g., scraping off corrosion products from the sample). Instead, the sample is placed directly in the spectrometer and a spectrum can be acquired.

When comparing IR spectra obtained by the different sub-techniques described above, it is necessary to be aware of that spectral difference may appear both concerning relative peak intensities as well as central wavelength. Comparing transmission and ATR spectra there may be differences in relative peak intensities since the penetration depth in the ATR mode depends on the wavelength of the IR radiation. Moreover, frequently a blue shift in IRRAS spectra of a peak position is observed when comparing to transmission spectra, which is due to the fact that the shape of IRRAS spectra not only depends on the absorption constant but also the real part of the refractive index [6]. For example, for the common corrosion product Cu_2_O on copper, a shift from 623 (transmission) to 655 (IRRAS) cm^−1^ was observed [6].

#### 2.1.2. IR Transmission Spectroscopy

IR transmission spectroscopy has been used quite rarely to identify atmospheric corrosion products, probably due to the difficulties in the sample preparation, which requires that the corrosion products are scraped off from the surface and pressed into a pellet together with for example KBr as discussed above. Thus, tiny amounts of corrosion products are difficult to study with this technique. In some examinations the IR studies have been complemented by for example XRD, in order to confirm the nature of the corrosion products.

##### 2.1.2.1. Steel

A number of studies on various steels have been performed, and the IR spectra have been used to identify the different phases of FeOOH, which have different characteristic infrared absorption bands. Additionally, the presence of bound water in the film of corrosion products has been concluded in some studies. Wang et al., studied the atmospheric corrosion of carbon steel during 12 months near Qinghai salt lake in China. Based on the results obtained from IR transmission spectroscopy it was concluded that β-FeOOH (450, 690, and 840 (weak)·cm^−1^), γ-FeOOH (1020 cm^−1^), and δ-FeOOH (790, 880, and 1110 cm^−1^) were formed, where the latter peak could be used to qualitatively assess the crystallinity since less crystalline samples exhibited a broader band [7,8]. In addition, water in the samples was revealed by a peak at 1630 cm^−1^ (bending vibration). The corrosion product Fe_8_(O,OH)_16_Cl_1.3_ was observed by XRD but those results could not be verified by IR spectroscopy due to the lack of reference spectra.

In a study of carbon steel by Allam et al., IR spectroscopy and X-ray diffraction were used to characterize the corrosion products formed during a 12-month exposure in an industrial atmosphere near the sea. The results indicated that the corrosion started by the formation of blisters containing iron chlorides, oxyhydroxides, sulphates, oxides, and possibly hydroxides [9]. Jaen et al., used IR spectroscopy in combination with Mössbauer spectroscopy to study the atmospheric corrosion of mild steel (A-36) and weathering steel (A-558 and COR 420) during three-month exposure to a tropical marine climate in Panama. They could identify the phases α-FeOOH and γ-FeOOH on all steels, but no δ-FeOOH was observed. In a similar way (although based on different peaks) as Wang et al., above concluded that the rust was of low crystallinity, the peaks in the study by Jaen at 790 and 884 cm^−1^ were weak and broad for the A-36 and A-558 samples. Hence, this indicated low crystallinity of the corrosion products. In contrast, the COR 420 showed highly crystalline α-FeOOH and γ-FeOOH. In an atmospheric corrosion study of pre-corroded (in SO_2_, NO_2_, and H_2_S) carbon steel at various locations in China, IR spectroscopy was used to identify α-FeOOH, γ-FeOOH, and δ-FeOOH in both rust flakes and powder-like rust. Strong OH stretching vibrations at 3120 and 3380 cm^−1^ were characteristic of γ-FeOOH and δ-FeOOH, respectively. Further, the corrosion products contained large amounts of water, indicated by the peak at 1640 cm^−1^, similarly to the rust studied by Wang et al., above.

Raman et al., used transmission IR to identify different kinds of rust on samples from outdoor environments, and present spectra from several different rust types as well as tables with characteristic infrared absorption bands [10]. A general conclusion is that crystalline corrosion products exhibit well defined peaks, whereas more amorphous products yielded weaker and broader bands.

Pacheco et al., examined the rust formation in chloride rich environments with different relative humidities, and observed the formation of oxides and oxyhydroxides during early corrosion [11].

##### 2.1.2.2. Copper

Morcillo et al., identified several corrosion products formed during exposure of copper samples in Ibero-America, the main product being Cu_2_O [12], as well as the corrosion products formed on zinc in rural and urban atmospheres [13]. The atmospheric corrosion products of the seven meter long iron cannon in Tanjore (India) were characterized by the use of IR spectroscopy [14].

##### 2.1.2.3. Zinc

Transmission IR spectroscopy of corrosion products and reference samples was used to identify the corrosion products formed during dry/wet cycles of zinc first exposed to a NaCl solution and then to humid air and CO_2_(g) [15]. The details of the studies are found in the IRRAS section.

##### 2.1.2.4. Aluminum and Magnesium Alloys

Ke et al., investigated the atmospheric corrosion of the aluminum alloy AA2024 in the presence of magnesium chloride-based multicomponent salts [16]. IR transmission studies revealed, in agreement with XRD examinations, that longer laboratory exposures resulted in corrosion products with a higher crystallinity than those formed in shorter exposure times, with the main corrosion products being [Mg_1_^_^*_x_*Al*_x_*(OH)_2_]*^x^*^+^ Cl^−^*_x_*·*m*H_2_O. The presence of the salts could induce corrosion also below 30% RH. IR transmission was used to characterize the atmospheric corrosion products formed on the magnesium alloy AZ91D exposed in a polluted environment [17]. FTIR in combination with XRD enabled the identification of Mg(OH)_2_, MgCO_3_, and Mg_2_Al_2_(SO_4_)_5_·39H_2_O as corrosion products, and it was further concluded that the corrosion rate was higher for an ingot sample in comparison to a die-cast sample. The corrosion was initiated in the α phase, being less noble than the β phase, which remained and constituted a barrier to further corrosion.

##### 2.1.2.5. Corrosion Inhibitors

In one study, transmission IR coupled with thermogravimetric analysis was used to examine model compounds that simulate amine-carboxylic acid-based volatile corrosion inhibitors, which for example have been used in the protection of mild steel against atmospheric corrosion [18]. IR measurements of the vapor evaporated from mixtures of two-component equimolar solutions were acquired, and it was concluded that initially the vapor contained essentially only free amine. However, the vapor phase composition changed over time and it was thus concluded that great care must be taken when using this type of mixtures as corrosion inhibitors.

#### 2.1.3. Diffuse Reflectance (DRIFTS)

##### Nickel

The atmospheric corrosion of nickel surfaces exposed outdoors at 39 test sites in European and North American countries was studied by DRIFTS [19]. The measurements were complemented by investigations where XPS, X-ray powder diffraction, and elemental analysis were used, and an agreement was found between the different methods. The identified corrosion products were sulfates characterized by strong bands at 600 and 1100 cm^−1^, water and hydroxides in the region 3000–3600 cm^−1^, and indications of water was also seen through the bending vibration at 1595 and 1600 cm^−1^, as well as libration modes in the region 660–900 cm^−1^. Two types of samples were identified, where the first type was non-crystalline and exhibited a lower protective ability, and showed distinct antisymmetric sulfate stretching bands at 1080, 115, and 1150 cm^−1^ as well as three hydroxyl peaks around 3600 cm^−1^. The high wavenumbers indicate that these hydroxyl groups possess very weak hydrogen bonds. The other type of samples had broader and unresolved sulfate and hydroxyl bands, showed a higher resistance towards corrosion, and were formed at longer exposure times. It was suggested that the spectral differences were due to different particle sizes, which is known to affect the band widths in DRIFTS or that the broader bands signified a more disordered product [20]. Also the water bending mode displayed different properties, with the band at 1660 cm^−1^ being strongest for the first type and the band at 1959 cm^−1^ for the second type.

#### 2.1.4. Infrared Reflection/Absorption Spectroscopy (IRRAS)

IRRAS is the infrared technique that most frequently has been utilized to study corrosion products formed under atmospheric conditions. The technique is well suited to identify the nature of corrosion products and their kinetics of formation. Preferably the metal surface should be shiny, since a dull and rough surface results in a diffuse reflection of the IR beam from the surface and hence little light that reaches the detector. If calibrated to a mass sensitive instrument such as quartz crystal microbalance (QCM), IRRAS can additionally provide information about the absolute mass of the corrosion products, as discussed below.

A problem frequently encountered when performing long time in-situ exposures is a change in the background signal, for example by drift or a varying relative humidity. Such changes can cause broad water bands to overlap with the peaks of interest, and hence make the interpretations difficult. A solution to this problem is to use polarization modulated (PM) IRRAS, in which the polarization is modulated between s and p-polarized light (s denotes light polarized perpendicular and p parallel to the plane of incidence), where s-polarized does not give rise to any surface signal due to a cancellation of the electric field in the surface region, whereas the p-polarized is enhanced in the surface region. A further advantage of PM-IRRAS is the improved signal to noise ratio. Some PM-IRRAS studies are discussed below. IRRAS has also been employed to study the properties of ultrathin organic films used as corrosion inhibitors, as elaborated in this section.

##### 2.1.4.1. Iron

Weissenrider at al. studied the atmospheric corrosion of iron induced by relative humidity, SO_2_(g), NO_2_(g), and O_3_(g) in-situ by IRRAS, QCM, and AFM [21]. It was concluded that NO_2_(g) or O_3_(g) in addition to SO_2_(g) were necessary to form sulfate nests on the iron surface. By the use of H_2_O as well as D_2_O, a band at 1100 cm^−1^ in the IRRAS spectra was identified as an OH vibration of some nature.

Kotenev et al., employed IRRAS, resistometry, and gravimetry to study the oxidation of metal oxide nanocomposite layers on sensors [22]. By identifying the characteristic peaks of different phases, it was concluded that Fe_3_O_4_ (412 cm^−1^), α-Fe_2_O_3_ (555 and 602 cm^−1^), and γ-Fe_2_O_3_ (652 cm^−1^) were initially formed, whereas after longer time α-Fe_2_O_3_ dominated. Thus, at the initial stages (5–30 min), the metal oxide structure contains Fe_3_O_4_, α-Fe_2_O_3_, and γ-Fe_2_O_3_, while at 2–5 h of oxidation, α-Fe_2_O_3_ is mainly present.

Aramaki prepared iron surfaces covered with a monolayer of 11-mercapto-1-undecanol, and on top a monolayer of bis(triethoxysilyl)ethane and octadecyltriethoxysilane to study the protection against atmospheric corrosion [23]. The film was characterized by XPS, contact angle, and IRRAS, and the conclusion was that the thiol formed a polymeric structure with the silanes, with siloxane bridges in the lateral direction. The nature of the corrosion products was characterized by XPS after exposure to air.

Stratmann and coworkers used IRRAS and quartz crystal microbalance to study the formation of ultrathin polysolixane layers on iron surfaces, as well as the protection ability of the films [24]. They found that the films significantly improved the corrosion resistance in a humid atmosphere containing SO_2_(g), and that the corrosion started at local defects in the film, but no changes in the polymer film itself were observed.

##### 2.1.4.2. Steel

Person et al., used IRRAS to study the atmospheric corrosion of Zn-Al-Mg coated, electrogalvanized, and hot dipped galvanized steel with deposited NaCl and exposed to 805 RH and 350 ppm CO_2_ [25]. In complement, XRD and SEM were used. During exposure NaCl(s) adsorbs water and a thin electrolyte layer is formed, which promotes the corrosion. With IRRAS several bands originating from water and hydroxide were observed at early stages (H_2_O and OH^−^ stretching vibrations in the region 3000–3700 cm^−1^), the water bending vibration at 1635 cm^−1^, and several bands in the region 400–1000 cm^−1^. Later the antisymmetric stretch of the carbonate ion was observed as a symmetric peak centered at ~1385 cm^−1^, which altogether indicated that layered double hydroxide products with intercalated carbonate ions had formed. Upon introduction of dry air, the water bands got reduced, an indication of that water left the surface. Complementary ATR spectra of reference compounds were acquired to assist in the assignments of the vibrations.

Kim and coworkers studied how steel could be protected against atmospheric corrosion (10 min in air at 400 °C and at 60 °C and 100% RH for five days) by covering it with silane primers and an epoxy layer, where the silane used to improve the adhesion of the epoxy layer to the metal surface [26]. The silane reacted with the steel surface to form a Fe-O-Si bond. IRRAS was used to conclude that upon heating of the epoxy covered steel surface, the epoxide peak at 915 cm^−1^ vanished, an indication that the epoxy film was completely cured. For both exposure conditions the corrosion was reduced, but the corrosion products Fe_3_O_4_, γ-FeOOH, and Fe_2_O_3_ could be observed in various amounts depending on the conditions.

##### 2.1.4.3. Copper

Person et al., used ex-situ IRRAS complemented by Raman spectroscopy, cathodic reduction, and XPS to examine the atmospheric corrosion of copper exposed to 75% RH and 0.25 ppm SO_2_(g) and NO_2_(g) [27]. Copper sulfite and sulfate were identified by IRRAS and a comprehensive table summarizing the band positions for sulfite and sulfate was presented. The spectra indicated the formation of a 1 nm thick film of sulfate or nitrate. Differences in peak position in comparisons with transmission and IRRAS spectra were also discussed, and exemplified by a blue shift of 28 cm^−1^ for Cu_2_O in the IRRAS spectra compared to transmission, as also was discussed by Greenler [6]. No Cu-O-H bending vibrations were observed, an indication of that no basic copper sulfates were formed.

**Figure 1:**
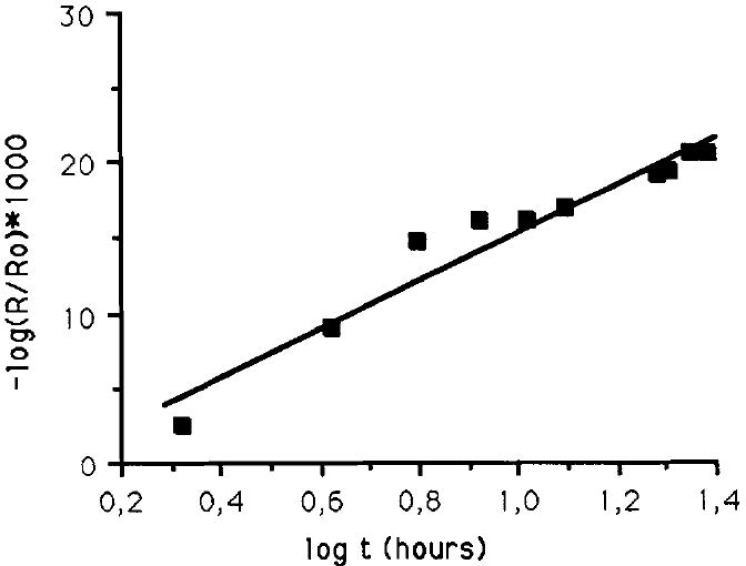
The infrared absorbance of the cuprite peak at 645 cm^−1^ as a function of exposure time when a copper surface was exposed to 90% RH, revealing a logarithmic oxide growth. Reproduced by permission of [28], The Electrochemical Society. Copyright 1993.

Person et al., later developed a sample cell for in-situ IRRAS studies of atmospheric corrosion processes, with the ability to control the relative humidity [28]. It was shown that during exposure of a copper surface to 90% RH, Cu_2_O was formed as revealed by an IR band at 645 cm^−1^. The kinetics of formation displayed a logarithmic behavior, indicated by a straight line when the IR absorbance of the cuprite peak was plotted against the logarithm of the exposure time, as shown in Figure 1. Hence, this demonstrates the possibility to use IRRAS to determine the kinetics of formation of corrosion products. To quantify the amount of cuprite formed, ex-situ cathodic reduction was used, and the amount of cuprite was further estimated through theoretical calculations. Further exposures to 80% RH and 0.23 ppm SO_2_(g) revealed the formation of sulfite by a band at 1010 cm^−1^, in addition to cuprite.

Kleber et al., also developed an in-situ IRRAS chamber and studied the formation of cuprite on a copper surface exposed to 80% RH [29]. As a complement, simulated spectra with corrosion film thickness and angle of incidence of the IR beam as variables were obtained, and AFM measurements to study the topography were performed. By plotting the IR absorbance of the cuprite peak as a function of time, it was concluded that the early copper corrosion follows a logarithmic rate.

In a subsequent study by Person et al., copper, zinc, and nickel were exposed to 0.21 ppm SO_2_(g) and 80% RH, and the formation of sulfite could in all three cases be followed from a few minutes after the start of the exposure to 20 h [30]. Through the IR peak positions of the sulfite bands it was possible to deduce the sulfite ion coordination on the three metal surfaces. A mechanism for the sulfur dioxide induced corrosion was also discussed, and estimations of the deposition rate of sulfur dioxide were done based on calculations and intensities of the sulfite bands. It was concluded that the deposition rate on zinc is one order of magnitude higher than that on the other metals, which indicates a surface reaction that is mass transport limited.

The same authors as above used IRRAS (complemented by XPS) to study field exposures of zinc, copper, nickel, and silver surfaces in indoor environments [2]. The locations were the Royal Palace in Stockholm, the St. Vitus cathedral in Prague, a military storage room in Karlstad (Sweden), and a computer room at a paper and pulp industry (Mönsterås, Sweden). The extreme temperatures altogether were −3° and 25°, and the relative humidity was in the range 17%–90% RH. These field exposures of course involve a significantly more complex atmosphere in comparison to the vast amount of model studies discussed in this article, and hence the peak assignments in the IR spectra become more difficult since numerous peaks are present and the nature of the atmosphere is more or less unknown. A table of important gases in indoor corrosion is found in Table 1 above. With IRRAS it was concluded that main corrosion products on zinc, copper, and nickel were carboxylates (e.g., formate and acetate), and that also tiny amounts of chloride, ammonium, nitrate, and sulfate ions were present. In contrast, silver sulfide was the main corrosion product on silver, and small amounts of sulfate, ammonium, and chloride ions were observed. Thicker layers of corrosion products were observed at the St. Vitus cathedral and in the military storage room, which was suggested to be due to a higher relative humidity and accordingly a more pronounced water layer on the metal surfaces. This study further shows the importance of organic corrosion products in indoor environments.

Aastrup et al., developed a sample cell which allowed simultaneous and in-situ studies by IRRAS and QCM of an atmospheric corrosion process of any relative humidity, hence yielding information about the nature of the corrosion products (IRRAS) and their mass (QCM) [31]. The capability of the setup was demonstrated by investigating the formation of cuprite when copper was exposed to 80% RH. By following the time evolution of the peak intensity of the cuprite band at 645 cm^−1^ and changes in QCM frequency, the kinetics of formation of the corrosion process could be monitored. The detection limit for IRRAS was a film thickness of 10 Å and for QCM 2 Å. The kinetics of formation was compared with cathodic reduction results, showing that all results were in agreement with each other. Itoh et al., also developed a combined in-situ IRRAS/QCM setup and measured the initial atmospheric corrosion of copper exposed to 80% RH and 10 ppm SO_2_(g) [32]. Sulfite (1040 cm^−1^) and sulfate (1110 cm^−1^) were identified as corrosion products, and complementary XPS studies showed the presence of both Cu(I) and Cu(II) oxides. Water in the film was revealed by bands at 3350 (stretches) and 1640 (bending mode)·cm^−1^. Both changes in the IR absorbance and the QCM mass indicated a parabolic growth of the corrosion products.

Wadsak et al., studied the atmospheric corrosion of copper in humid air of 60% and 80% RH using a coupled setup for IRRAS and QCM, as well as AFM [33]. With IRRAS it was concluded that Cu_2_O (645 cm^−1^) formed for both relative humidities, and AFM enabled a determination of tiny corrosion products at 60% RH and a more fully covering layer at 80% RH after 80 min. With QCM it was possible to conclude that the corrosion rate initially was fastest in both cases and that the mass gain was slower at the lower relative humidity.

**Figure 2:**
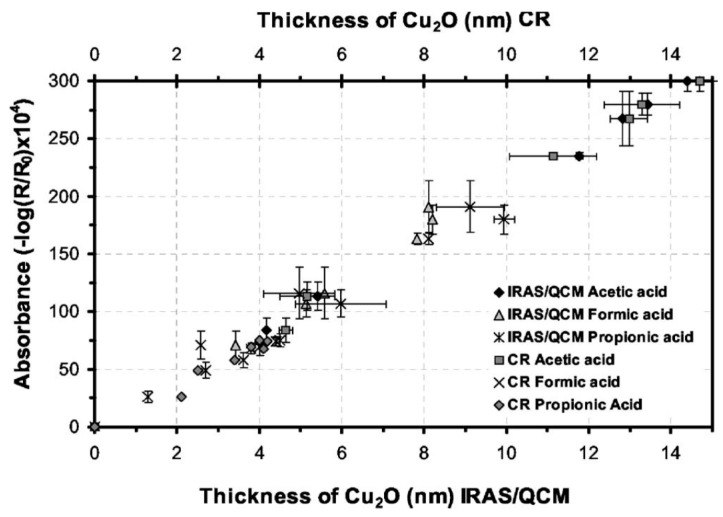
A linear relation between the results obtained from infrared reflection/absorption spectroscopy (IRRAS), quartz crystal microbalance (QCM), and cathodic reduction for the formation of Cu_2_O during exposure of a copper surface to 90% RH and 120 ppb carboxylic acids. Reproduced by permission of [35], The Electrochemical Society. Copyright 2007.

Gil, et al., published a series of articles where the atmospheric corrosion of copper was studied by combining IRRAS and QCM, to obtain the nature of the corrosion products as well as their mass. A combination of three analytical techniques, IRRAS, cathodic reduction, and QCM allowed a detailed study of the initial atmospheric corrosion of copper exposed to 90% RH and either 120 ppb formic, acetic [34], or propionic acid [35]. With IRRAS the corrosion products cuprite, copper carboxylates, and copper hydroxide as well as water in the film were identified. A linear correlation between the IR absorbance of the cuprite peak at 648 cm^−1^, the mass of cuprite obtained from QCM, and the thickness of the cuprite layer as determined by cathodic reduction was obtained (Figure 2), thus providing a convincing conclusion about the formation of corrosion products. Correlating the mass of the corrosion products obtained by QCM with the absorbance from IRRAS allows future determinations of the mass of corrosion products to be obtained solely by the use of IRRAS, since the absorbance scales linearly with the amount of corrosion products formed. Further, the growth of the copper carboxylate was followed by the absorbance of the antisymmetric carboxylate stretch at around 1600 cm^−1^. In complement AFM was used to determine the morphology of the corrosion products, and a mechanism for the reaction route was suggested. The IRRAS and QCM results were also compared with computer simulations using the GILDES model [36], which could predict the more aggressive corrosion induced by formic acid, followed by acetic acid and propionic acid [36]. The trend was explained by a higher ligand-, and proton promoted dissolution for formic acid.

Kleber et al., combined in-situ IRRAS, in-situ AFM, and SIMS to investigate the atmospheric corrosion of copper, zinc, and two brass alloys (Cu/Zn = 70/30 and 90/10 wt %) exposed to 80% RH and 250 ppb SO_2_(g) [37]. IRRAS revealed the formation of Cu_2_O when copper was exposed to humid air, and by integrating the cuprite peak a logarithmic rate for cuprite formation was observed. Although Zn(OH)_2_ likely is formed in the presence of water, the bands could not be observed due to overlap with the bands from gas phase water. For copper and 90/10 brass a cuprite peak at 650 cm^−1^ was observed. Its absence for 70/30 brass indicated that only zinc corroded. However, no reliable signal from ZnO at 500–550 cm^−1^ could be detected due to the cutoff frequency of the MCT detector. With AFM it was concluded that the brass alloys exhibited larger corrosion products than the pure metals. By a comparison of the intensities of bands originating from metal sulfides, sulfites, and sulfates it was concluded that the brass alloy with the highest zinc content corroded faster in the presence of SO_2_(g). As in the exposure of only humid air, Cu_2_O was only observed for copper and 90/10 brass.

Faguy et al., developed a setup for in-situ PM-IRRAS measurements, and showed that their PM-IRRAS improved the signal to noise ratio in comparison with conventional IRRAS with a factor of 2.5 [38]. It was furthermore possible to obtain spectra without interference from gas phase water. The capacity of the technique was demonstrated in studies of copper exposed to SO_2_(g), NO_2_(g), and HCl(g), and nitro, nitrito, and sulfate corrosion products were identified, and the kinetics of formation could be followed.

Malvault et al., synthesized a number of basic copper salts, brochantite (Cu_4_SO_4_(OH)_6_), gerhardite (Cu_2_NO_3_(OH)_3_), atacamite (Cu_2_Cl(OH)_3_), aratacamite (Cu_2_Cl(OH)_3_), malachite (Cu_2_CO_3_(OH)_2_), and posnjakite (Cu_4_SO_4_(OH)_6_·H_2_O), to use as reference compounds for interpretations of IRRAS spectra of corroded copper surfaces [39]. Additionally, cathodic reduction was used to characterize the corrosion products. Interestingly, in contrast to Cu_2_O [6], the band positions in the IRRAS and transmission spectra were not shifted.

The atmospheric corrosion of copper covered with sodium chloride and exposed to either CO_2_(g) [40] or SO_2_(g) [41] was studied by Chen et al., and is discussed further under the section Conventional IR microscopy, Section 2.1.6.1.

By the use of in-situ IRRAS and two-dimensional correlation analysis (2D-IR), the Osawa group determined the nature of the corrosion products as well as their kinetics of formation when a copper surface was exposed to 80% RH and 8.7 ppm SO_2_(g) [42]. The 2D analysis facilitated a deconvolution of the infrared bands, and was used to follow the kinetics of formation of the main corrosion products CuSO_3_Cu_2_SO_3_·2H_2_O and CuSO_4_·5H_2_O. Initially a water adlayer was formed on the surface, and subsequently the sulfur containing corrosion products were formed.

IRRAS has been used a number of times to examine the quality of ultrathin corrosion inhibiting films, in addition to studies of corrosion products. Mekhalif and coworkers prepared self-assembled monolayers of flurothiols on copper and used IRRAS and XPS to characterize their structure and organization, and studied their protection by cyclic voltammetry [43]. Liedberg and coworkers investigated the chemisorption of the corrosion inhihitor benzotriazole on copper and cuprous oxide with IRRAS, X-ray photoelectron spectroscopy, and ultraviolet photoelectron spectroscopy [44]. IRRAS was used to study variations in the orientation of the benzotriazole molecules in the layers, and the authors further suggested a new orientation of this inhibitor on copper. The Ishida group studied the ability of imidazoles to protect copper surfaces against corrosion in a series of articles [45,46,47]. IRRAS was used to examine the molecular structure, orientation, and stability of the inhibitors prepared under different conditions and exposed to various heat treatments. Jang and coworkers used IRRAS and scanning electron microscopy to study the effect of heat treatments on protective films of various compositions of a copolymer consisting of vinyl imidazole and vinyl trimethoxy silane deposited on copper [48]. The authors observed that by the introduction of latter polymer, the heat resistance was improved due to the formation of disolixane linkages. Wenger and coworkers investigated the thermal stability of imidazole and 5-methylbenzimidazole films on copper surfaces in atmospheres containing nitrogen gas, air, or under vacuum conditions [49]. The authors concluded that the organic layers under heat treatment were more stable in nitrogen and vacuum in comparison to air, due to oxidation. The decomposition of the prohibiting films was monitored by plotting the IR intensity of a vibration in the organic molecules and following the change in time.

##### 2.1.4.4. Silver

**Figure 3:**
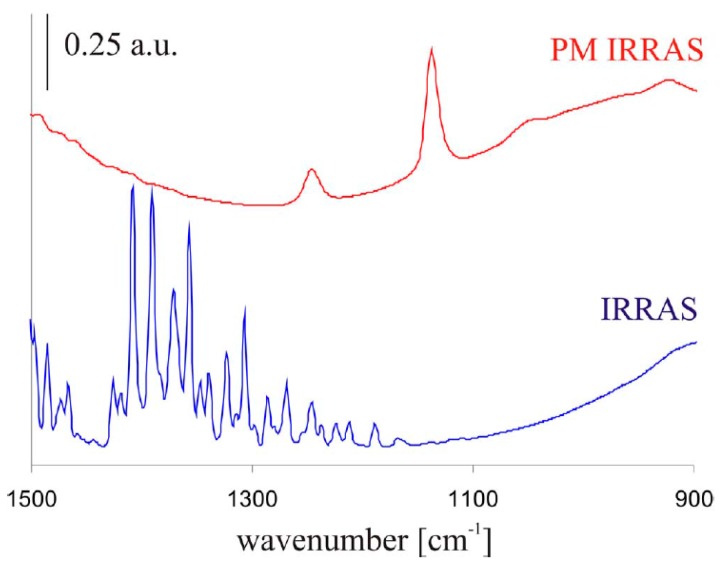
A comparison of PM-IRRAS and IRRAS to detect thin layers of corrosion products, revealing the clear benefit of using PM-IRRAS. Reprinted from [50] with permission from AIP Publishing. Copyright 2014.

In an atmospheric corrosion study by Wiesinger et al., of a silver surface exposed to 90% RH and 500 ppm CO_2_, the ability of IRRAS and PM-IRRAS to detect corrosion products was compared [50]. As shown in Figure 3, PM-IRRAS was superior in detecting weak peaks and cancelling the effect of water vapor.

The same research group used conventional IRRAS in combination with QCM to study the formation of basic carbonates on a silver surface under the influence of UV light under in-situ conditions [51,52]. IRRAS enabled the nature of the corrosion products to be determined, and QCM their mass. The bands at 922 ([HO-CO_2_]^−^ skeletal vibration), 1109 (asymmetric—C-O stretch), and 1221 (antisymmetric CO_3_^2−^ stretch) cm^−1^ were used to identify the formation of AgOHAg_2_CO_3_. The growth of these bands with time indicated a continuous formation of basic silver carbonate. Irradiating the sample with UV light during the exposure significantly enhanced the infrared bands corresponding to the corrosion products, and accordingly an enhanced corrosion rate. Broad bands above 3000 cm^−1^ indicated the presence of physisorbed water.

Further studies include investigations of the initial atmospheric corrosion of silver, iron, and copper, as investigated by IRRAS, QCM, AFM (in-situ), and XPS [53]. Under exposure to 90% RH, the IRRAS results indicate a continuous formation of cuprite (~650 cm^−1^), whereas no corrosion products were observed at the iron surface, only physisorbed water. XPS indicated the formation of silver oxide and silver hydroxide. The thickness of the surface water layer, which greatly affects the corrosion rate, increased for a certain relative humidity in the order Fe < Cu < Ag, of which copper however displayed the most pronounced corrosion. Upon addition of SO_2_(g), CuSO_3_ was detected as islands, but no sulfur containing species were detected on silver, only an increased corrosion rate. For iron, still no corrosion was observed, and a requirement to induce atmospheric corrosion of iron appears to be that also NO_2_(g) is added.

##### 2.1.4.5. Zinc

**Figure 4:**
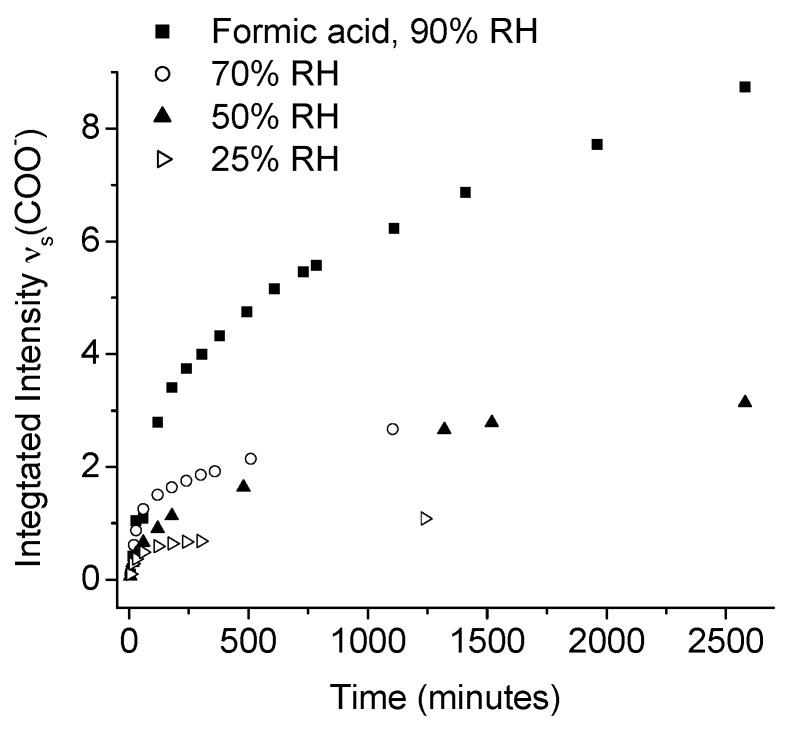
Zinc exposed to formic acid at different relative humidities. The x-axis shows the time and the y-axis the amount of zinc formate formed, as determined by the intensity of the antisymmetric formate stretch. Reproduced from [54] by permission of The Electrochemical Society. Copyright 2006.

Johnson et al., used IRRAS to study the atmospheric corrosion of zinc induced by the important indoor corrosion promoters formic (80 ppb) and acetic acid (100 ppb), as well as acetaldehyde (80 ppb), with a relative humidity of 90% [54,55,56]. Through the IRRAS data it was concluded that zinc carboxylates were formed during all three exposure conditions, and that the initial corrosion was fastest, followed by a lower rate, as indicated by comparing the absorbance of the antisymmetric carboxylate stretching vibration. The reduced corrosion rate by time is an indication that the formed corrosion products acted as a partly inhibiting layer. It was further concluded that the corrosion rate increased with an enhanced relative humidity for all three systems, since the water adlayer on the zinc surface was more pronounced at a higher humidity. This is shown in Figure 4, which clearly shows that the corrosion is faster at a higher relative humidity.

In the exposure to formic acid, it was observed that the antisymmetric formate stretching vibration around 1630 cm^−1^ was blue shifted (and broadened) when the humidity was changed from 90% to 0% RH, an indication of a change in the environment when water is removed and the interactions between zinc formate and water are reduced. The absence of a peak (C=O vibration) above approximately 1690 cm^−1^ revealed that no or low amounts of physisorbed formic and acetic acid were present, but rather formate and acetate dominated. By studying the difference in wavenumbers between the symmetric and antisymmetric stretching vibrations it was concluded that the zinc acetate species formed a chelating bidentate or bridging structure. Since both the symmetric and antisymmetric carboxylate stretching vibrations possessed a significant intensity in all three systems studied, it was concluded that the zinc carboxylate species formed were randomly oriented. Complementary SEM examinations revealed that the corrosion products formed during acetic acid exposure had a radial growth, whereas acetaldehyde resulted in filiform corrosion [55]. Additionally, the role of the air/water interface in the above mentioned corrosion processes was investigated by VSFS, as discussed below.

The atmospheric corrosion of zinc was further studied by IRRAS by Qiu et al., in order to investigate the behavior of zinc exposed to 90% RH and either 120 ppb of formic, acetic, or propionic acid [57]. In order to deduce the mass of the corrosion products formed (ZnO at 570 cm^−1^ and zinc carboxylates with main peaks originating from carboxylate stretching vibrations in the region 1345–1620 cm^−1^), an optical model was used, and the properties of the corrosion products were further characterized by SEM and grazing incidence X-ray diffraction (GIXRD). The wavenumber difference in IR peak position between the symmetric and antisymmetric carboxylate stretching vibrations was used to conclude that the zinc formate formed a monodentate structure (Δν = 275 cm^−1^), whereas exposure to acetic acid and propionic acid resulted in a chelating or bidentate structure (Δν = 150 cm^−1^) [58].

**Figure 5:**
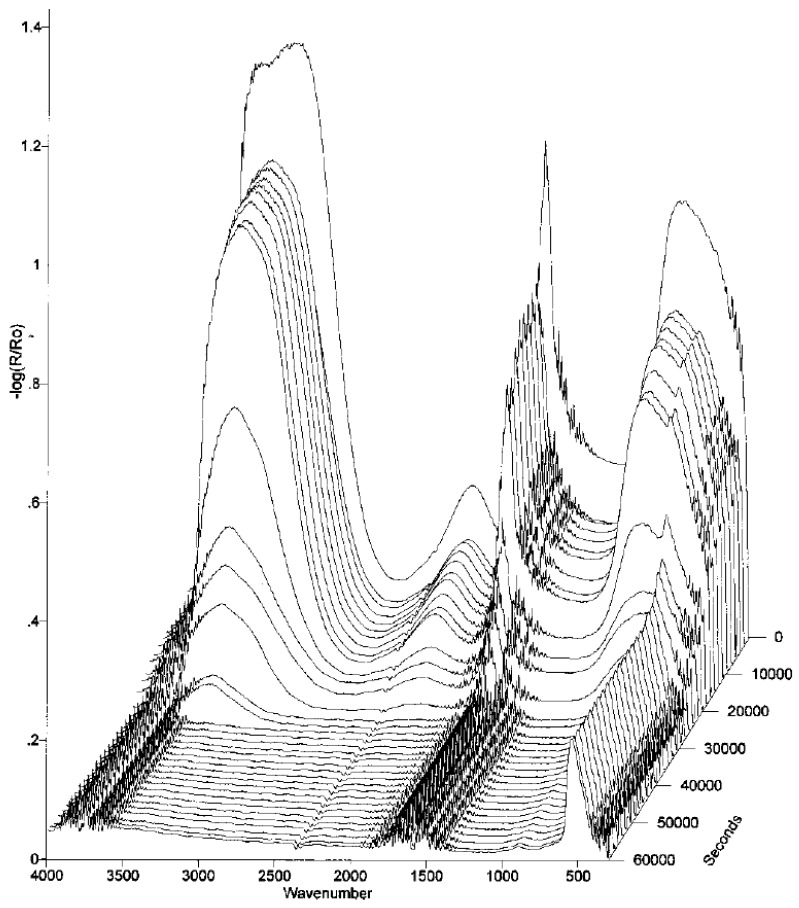
A series of IRRAS spectra acquired during the first drying phase of a zinc surface. The reduction of the water bands (~1645 and 3000–3600 cm^−1^) shows the removal of physisorbed water at the surface. Reproduced from reference [15] by permission of The Electrochemical Society. Copyright 2001. The y-axis shows the absorbance given as −log(R/R_0_), where R is the reflected radiation from the sample and R_0_ from the background.

Zhu et al., developed a cell for periodic dry/wet exposures, and examined the corrosion of zinc samples dipped for 1 h in a 1% aqueous NaCl solution, followed by a drying period of 23 h in 50% RH and a carbon dioxide concentration of <5 ppm or >350 ppm [15]. By following the intensity of the infrared bands (water bend at 1645 cm^−1^ and water stretches at 3000–3600 cm^−1^) originating from physisorbed water it was possible to follow the kinetics of the drying process, and after 6–8 h in 50% RH an equilibrium was reached. With CO_2_(g) concentrations below 5 ppm the dominant corrosion product was ZnO (560 cm^−1^) and small amounts of Zn_5_(OH)_8_Cl_2_^.^H_2_O (740 and 920 cm^−1^) was additionally observed. In contrast, with carbon dioxide concentrations above 350 ppm, the corrosion products Zn_5_(OH)_6_(CO_3_)_2_ (840, 730, 1040, 1300–1600, and 3400 cm^−1^) and Zn_5_(OH)_8_Cl_2_^.^H_2_O were detected. In-situ IRRAS spectra during the first drying phase with a carbon dioxide concentration below 5 ppm is seen in Figure 5.

In order to obtain information about both the nature of the corrosion products formed and the changes in Volta potential, IRRAS was combined with Kelvin probe measurements by Person et al., [59]. Zinc was exposed to humid air and by IRRAS ZnO, Zn_5_(OH)_8_Cl_2_·H_2_O and zinc hydroxy carbonate were observed as the corrosion products. Formation of these corrosion products was accompanied with an increase in Volta potential as a result of an enhanced inhibition of the film formed. In addition, IRRAS spectra of the corrosion products were simulated.

Kleber et al., studied the atmospheric corrosion of zinc, copper, and brass exposed to humid air and SO_2_(g), as described in Section 2.1.4.3 [37].

##### 2.1.4.6. Brass

Qiu et al., studied the atmospheric corrosion of brass (Cu-20Zn) in 90% RH by IRRAS, and complemented the studies by SEM/EDS, AFM, cathodic reduction, confocal Raman microscopy, and scanning Kelvin probe force microscopy (SKPFM) [60]. The formation of ZnO and Cu_2_O was revealed by peaks at 570 and 660 cm^−1^, respectively. By measuring the intensities of these bands as a function of time, it was possible to follow the kinetics of formation of the two corrosion products. As is described later, confocal Raman microscopy indicated that ZnO formed islands on a more fully covering cuprite layer. The same authors further studied the corrosion of brass exposed to formic, acetic, and propionic acid, and found by IRRAS that ZnO, Cu_2_O, Cu(OH)_2_ were formed [61]. To obtain more information about the corrosion products, XRD was used, and it was concluded that in the case of brass exposed to formic acid, Cu(OH)_2_·H_2_O and Zn(HCO_2_)_2_·*x*H_2_O were formed, whereas for acetic acid Zn_5_(OH)_8_(CH_3_CO_2_)_2_·*x*H_2_O was formed, but no crystalline oxide was observed. For propionic acid, no XRD peaks were observed, and hence the amount of crystalline products if any was below the detection limit.

As discussed in Section 2.1.4.3 on copper above, Kleber et al., studied the atmospheric corrosion of two brass alloys (Cu/Zn = 70/30 and 90/10 wt %), zinc, and copper exposed to humid air and SO_2_(g) [37].

##### 2.1.4.7. Tin

Takeshi et al., studied the atmospheric corrosion of tin exposed to 80%–90% RH as well as SO_2_(g) and NO_2_(g) [62]. Exposure to sulfur dioxide resulted in no formation of corrosion products due to the inhibiting ability of the outermost tin oxide layer, whereas during exposure to nitrogen dioxide SnO (720 cm^−1^), SnO_2_ (610–670 cm^−1^), tin nitrate (830, 1350, 1390, 1450 cm^−1^), and hyponitrite (1040 cm^−1^) were formed. The growth of corrosion products was observed as an increase in the IR absorbance by time. The chemical characterization was carried out in-situ by IRRAS, and complemented by Raman and XPS examinations. No synergistic effects of the two gases were observed.

##### 2.1.4.8. Bronze

Wadsak et al., studied bronze exposed to 80% RH and 250 ppb SO_2_(g) with in-situ IRRAS, as well as AFM and XPS [63]. During exposure to only humid air, no cuprite was observed in the IRRAS spectra, but it was concluded that more water was physisorbed on bronze in comparison to copper. In fact, the corrosion on bronze was slower in comparison with copper due to the presence of lead oxide at the surface on bronze. The presence of hydroxide ions was revealed by a band at 1100 cm^−1^. In exposure to humid air and sulfur dioxide, copper sulfite (1055 cm^−1^) was observed and after 500 min exposure an infrared band originating from Cu_2_O at 650 cm^−1^ appeared. The corrosion products could hence form when the protective layer of lead oxide was destroyed. IRRAS data also revealed that more water was adsorbed at the surface in presence of SO_2_(g) compared to its absence. With AFM it was concluded that larger corrosion products were formed in the presence of SO_2_(g).

##### 2.1.4.9. Aluminum

The corrosion of aluminum surfaces covered with a sulfuric acid film formed from SO_3_(g) and H_2_O(g) at a pressure of 200 Torr was studied by Dai et al., [64]. The corrosion rate was observed to increase at higher relative humidities and higher deposition of sulfuric acid. From 3 to 240 min the peaks at 1103 cm^−1^ (SO_4_^2−^) and 2510 cm^−1^ (Al_2_(SO_4_)_3_) increased in intensity, signifying deposition of sulfuric acid and an ongoing corrosion. The presence of the bisulfate ion was also confirmed by infrared bands at 890, 1050, and 1245 cm^−1^.

#### 2.1.5. Attenuated Total Reflection (ATR)

Stratmann and coworkers covered an ATR crystal with a 10 nm layer of iron, and exposed it to methyl-, and butyltrimethoxy silane at various relative humidities, resulting in a polymeric film at the surface [65]. The adsorption could be monitored by the use of a quartz crystal microbalance. At higher relative humidities thicker films were formed. Subsequently they exposed the system to 98% RH and 15 ppm SO_2_(g), and investigated the inhibiting properties of the polymer film. It was shown that the film increased the induction time for a corrosion process to be initiated significantly.

Person et al., used ATR as a complementary technique to IRRAS, as discussed above [25].

#### 2.1.6. IR Microscopy

Infrared microscopy allows studies of the local distribution of corrosion products, which is highly important since a heterogeneous formation often occur. In conventional IR microscopy where the IR beam is focused at the surface, the spatial resolution is at the best some μm since the technique is diffraction limited. In contrast, novel IR microscopy techniques where the IR beam is focused on an AFM tip can improve the spatial resolution considerably, down to 10–20 nm. The latter type of technique has thus the potential to significantly provide more details in studies of atmospheric corrosion.

##### 2.1.6.1. Conventional IR Microscopy

**Figure 6:**
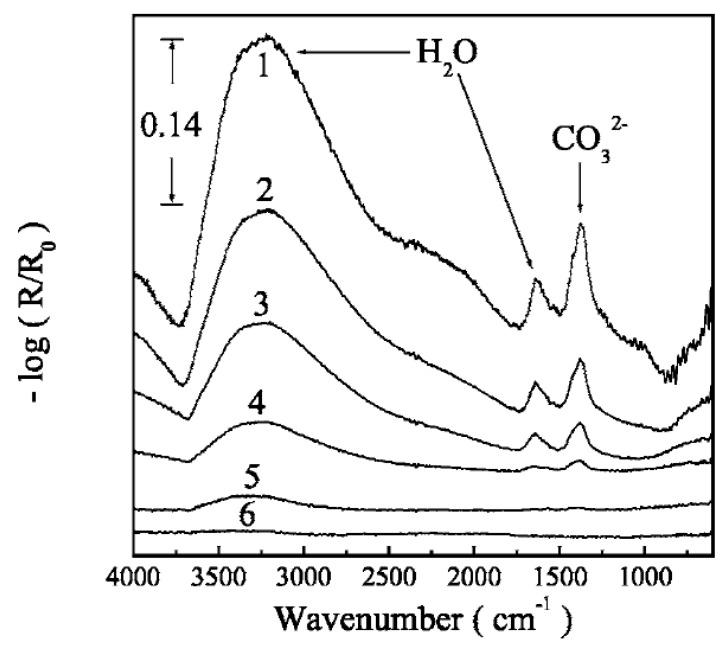
Infrared (IR) microscopy spectra taken at (1) −50; (2) −10; (3) 20; (4) 50; (5) 100; (6) 200 μm from the edge of the droplet [66]. A NaCl particle was deposited on the copper sample and it was exposed for 3 h to 350 ppm CO_2_(g) and 80% RH. Negative values indicate that the spots are within the droplet. Reproduced from reference [66] by permission of The Electrochemical Society. Copyright 2005.

The Leygraf group studied the atmospheric corrosion of copper with a single particle of sodium chloride deposited and exposed to 80% RH and different concentrations of carbon dioxide with IR microscopy (aperture size 100 × 100 μm), SEM, and Kelvin probe [66]. An electrolyte droplet formed around the salt particle and the corrosion products at different distances from the drop edge were examined by in-situ IR microscopy, as shown in Figure 6. The broad band centered at 3250 cm^−1^ indicates the presence of water and hydroxide groups, the band at 1640 cm^−1^ is the bending vibration of water, and the band at 1380 cm^−1^ reveals the formation of carbonate (stretching vibration). A thicker electrolyte layer closer to the edge of the droplet results in larger infrared bands, and the spectra indicate that carbonate was formed in a region less than 100 μm. In similar experiments but with a carbon dioxide concentration lower than 5 ppm, an additional peak at 3572 cm^−1^ appeared in certain spots, and was assigned to non-bonded OH groups, probably from a thin layer of Cu(OH)_2_.

Similar studies were also performed on copper with deposited sodium chloride exposed to 150 ppb SO_2_(g) and with either <5 ppm or 350 ppm CO_2_(g) [41]. Similarly, a water droplet was formed at the surface, which led to galvanic corrosion. In the area of the droplet, CuCl(nantokite), Cu_2_OH_3_Cl (paratacamite), sulfate, tiny amounts of carbonate, as well as S_2_O_6_^2−^ (dithionate) were formed, with the latter being observed for the first time in an atmospheric corrosion process. In the area defined by the secondary spreading of the droplet, sulfite, sulfate, and dithionate were observed.

**Figure 7:**
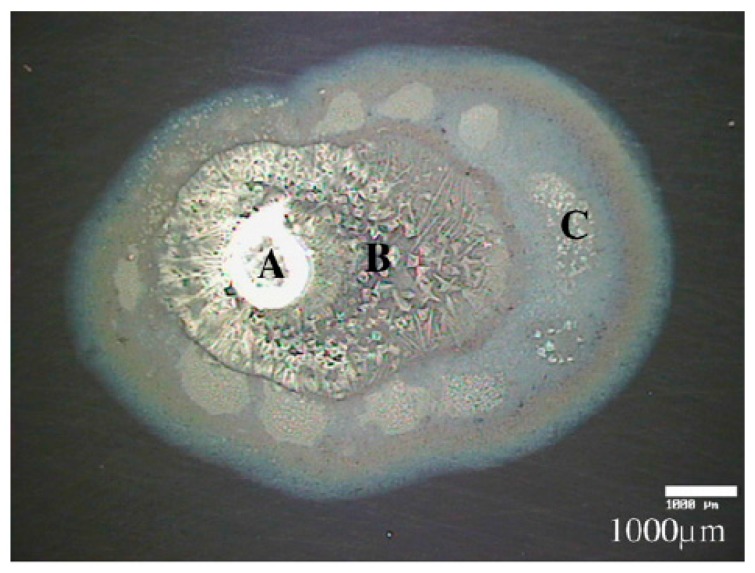
An optical image of a zinc surface with a deposited NaCl particle exposed to 90% RH for 6 h. The corrosion products identified in the three points A, B, and C are discussed in the text. Reprinted from reference [67] with permission from Elsevier. Copyright 2008.

The same research group studied the atmospheric corrosion of zinc with deposited sodium chloride exposed to SO_2_(g), CO_2_(g) and 80% RH [67]. IR microscopy with an aperture of 100 × 100 μm was used to qualitatively estimate the thickness of the electrolyte layer at various positions by comparing the intensity of the water bands at 1645 and 3400 cm^−1^. It was further possible to identify the corrosion products at different locations with ex-situ IR microscopy, as shown in Figure 7. In area A, the corrosion products consisted of Zn_5_(OH)_8_Cl_2_·H_2_O (simonkolleite) as revealed by peaks at 3470 and 3550 cm^−1^ from OH stretching vibrations, peaks at 727, 905, and 1044 cm^−1^ from Zn-O-H vibrations, and ZnO was identified by a band between 477 and 575 cm^−1^, whereas only a tiny peak originating from carbonate was observed at 1422 cm^−1^. In area B and C the corrosion products were sodium carbonate. At <5 ppm CO_2_(g), the corrosion products have a local character, whereas a more general corrosion was observed for 350 ppm CO_2_(g). Further, a slower spreading of the droplet formed at the sodium crystal particle was observed at the higher carbon dioxide concentration. For exposures to SO_2_(g), no secondary spreading of the droplet was observed. Bands between 1065 and 1192 cm^−1^ indicated the formation of sulfate ions, a formation that increased in the presence of sodium chloride. In contrast, no sulfite was observed.

**Figure 8:**
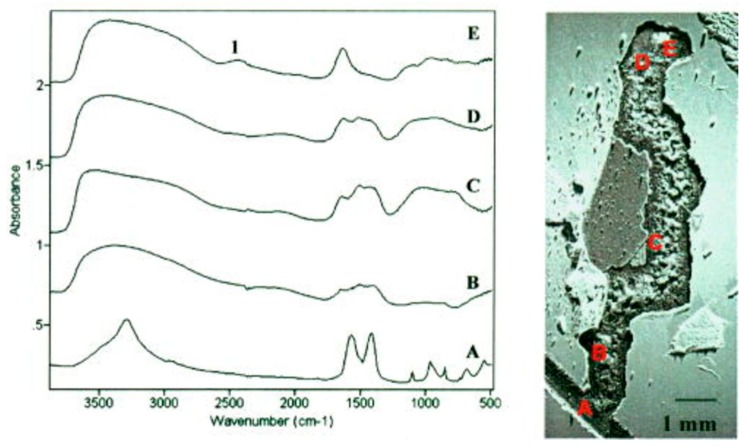
IR spectra obtained at the head and tail of the filiform corrosion products of the aluminum alloy AA6016 pretreated with Ti-Zr and covered with paint, exposed to 85% RH for six weeks [68]. Reproduced from reference [68] by permission of The Electrochemical Society. Copyright 2002.

Thierry and coworkers performed several IR microscopy studies of corroded aluminum and aluminum alloy surfaces. The AA6016 aluminum alloy coated by various methods (e.g., chromating and phosphating) was exposed to 85% RH at 25 °C for 6 weeks, and IR microscopy spectra were acquired with the aperture set to 250 × 250 μm [68]. The IR microscope allowed studies of the nature of the corrosion products both at the head and the tail of the filiform features, as shown in Figure 8. At the head (point and spectrum E), the corrosion products were identified as Al_2_(OH)_5_Cl·2H_2_O and/or Al(OH)_2_Cl, and probably an aluminum hydroxide gel. In contrast, in the tail (B, C, D), the corrosion products were identified as an aluminum hydroxy carbonate gel. In the scratch, dawsonite (crystalline sodium aluminum hydroxyl carbonate) was observed, a compound which forms at a pH in the range 7.5–9.5, meaning that an enhanced pH value must be prevalent in the scratch. It was anticipated that the presence of hydroxide ions was due to the reduction of oxygen. The IR microscopy measurements were complemented with IR transmission spectra of reference compounds, as well as with Volta potential measurements that revealed that the head had a potential 400 mV lower than the tail.

The initiation and propagation of the filiform corrosion of the same alloy covered with a polyurethane or polyester coating was also investigated by IR microscopy and Kelvin probe [69]. The sample was exposed to 85% RH and 350 ppm CO_2_(g). To study the initiation of the filiform corrosion, a scratch was made in the coating, NaCl was added, 85% RH was introduced, and IR spectra were acquired as a function of time. At the initial dry conditions, AlCl_3_ and Al(OH)_2_Cl were formed. The introduction of humid air results in the formation of a surface water layer, as revealed by broad bands in the region 3000–3600 cm^−1^. Moreover, in the initial period (5 min) Al(H_2_O)_6_^3+^ was identified through the peak at 2450–2500 cm^−1^, which was reduced in intensity after 1 h. At the same time, filaments started to form as the adhesion of the coating was reduced, a result of a low pH value and high chloride concentration. After longer exposures the peak from Al(H_2_O)_6_^3+^ vanished and carbonate peaks (bend at 850 cm^−1^, stretches at 1090, 1430, and 1520 cm^−1^) from an aluminum hydroxide gel containing carbonate appeared in the IR spectra taken in the scratch. The propagation of the filiform corrosion was further followed through the thin organic film with IR microscopy, and the movement of the head was followed by studying the peak from Al(H_2_O)_6_^3+^ at 2500 cm^−1^.

Extended investigations of the initiation and propagation of filiform corrosion on the same alloy were undertaken in order to study the effect of relative humidity, temperature, and wet-dry cycles [70]. The studies revealed that the filiform corrosion proceeded down to around 40% RH, the maximum filiform corrosion occurred at 75%–95% RH, an enhanced filiform corrosion was observed with an increasing temperature in the range 5–50 °C, and varying dry-wet cycle results were obtained depending on pretreatment.

Thierry and coworkers additionally studied the atmospheric corrosion of steel using IR microscopy. In one study the atmospheric corrosion of galvanized stainless steel covered with an epoxy resin was examined at a defect point at 90% RH [71]. Due to corrosion, de-adhesion of the coating occurred, and the corrosion products under the coating were identified by IR microscopy. It was concluded that in front of the de-adhesion simonkolleite was formed, whereas 1 mm away the main corrosion product was hydrozincite. Complementary scanning Kelvin probe measurements together with the IR microscopy measurements enabled the localization of the anode and cathode to be determined. IR microscopy was in addition used to identify anode and cathode areas on carbon steel suffering from atmospheric corrosion [72].

In a field study the distribution of corrosion products on nickel and zinc surfaces was examined by IR microscopy, and complemented studies with SEM/EDS were performed [73]. The corrosion products formed inhomogeneously over the surface and were dominated by Zn_4_CO_3_(OH)_6_·H_2_O, ZnSO_3_·*n*H_2_O, Zn_4_SO_4_(OH)_6_·*n*H_2_O, and NiSO_4_·*x*H_2_O.

##### 2.1.6.2. Nano IR Microscopy

**Figure 9:**
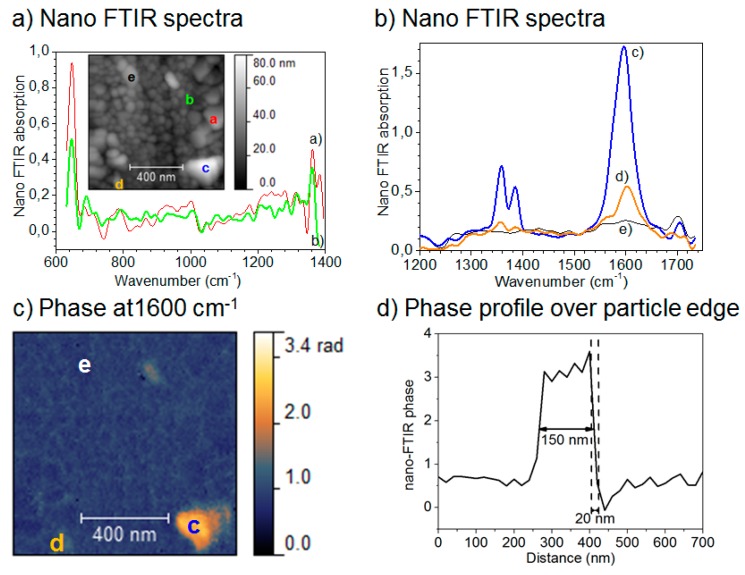
(a) Nano Fourier transform infrared spectroscopy (FTIR) spectra showing the presence of Cu_2_O at 650 cm^−1^ with an inset displaying an AFM topography map; (**b**) IR spectra a point (**c**–**e**) in figure (**a**,**c**) an IR phase image at 1600 cm^−1^ where the phase is related to the IR absorption; and (**d**) an IR profile over a particle showing a spatial resolution of 20 nm. Reprinted from reference [74] with permission from Elsevier. Copyright 2016.

Although conventional IR microscopy has provided a lot of important information about the distribution of corrosion products, it suffers from the problems that the spatial resolution due to diffraction limitation at the best is around 5 μm, as well as that μm-thick films are required to be observed. With the novel technique nano FTIR microscopy both these problems are resolved, and the nature of corrosion products can be determined with a spatial resolution of 20 nm. Johnson et al., studied the distribution of the corrosion products cuprite and copper formate at a copper surface exposed to 80% RH and 100 ppb formic acid, to simulate an indoor corrosion process [74]. IRRAS was initially used to determine the nature of the corrosion products over the whole surface, and nano IR to scrutinize the spatial distribution of corrosion products. Figure 9a,b shows infrared spectra at certain positions of the corroded surface, where the spectra in Figure 9a reveal the presence of Cu_2_O as by the peak at 650 cm^−1^, and the spectra in Figure 9b show spectra corresponding to copper formate at the points marked by “c” and “d”, whereas no copper formate was present at point “e”. The inset in Figure 9a is an AFM topography image that shows the positions of the points. Figure 9c shows an IR image acquired at 1600 cm^−1^, which is the peak position of the strongest copper formate peak in Figure 9b. The particle denoted as “c” gives a large contrast and is hence enriched in copper formate, which agrees with the spectrum in Figure 9b where the same location results in the strongest spectrum. Figure 9d shows an IR intensity profile (1600 cm^−1^) over a copper formate particle with a diameter of 150 nm, and the spatial resolution over the edge is 20 nm.

**Figure 10:**
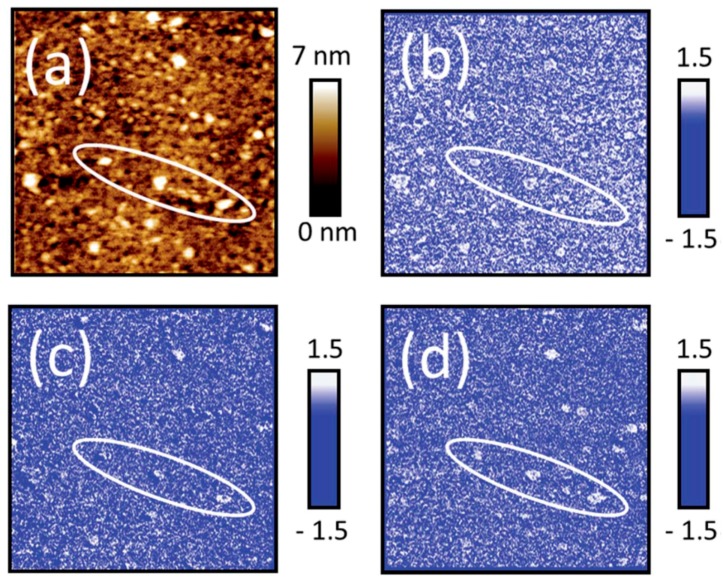
(**a**) Atomic force microscopy (AFM) topography image, amplitude images at the wavenumbers (**b**) 3420/2964; (**c**) 3296/2964; (**d**) 3296/3420. The circled regions are the ones discussed in the text. Reproduced from [77] with permission from the Royal Society of Chemistry. Copyright 2015.

Lyon and coworkers have used AFM-IR to study the heterogeneous structure in organic coatings as well as their water uptake [75,76,77]. In a study of a phenolic epoxy resin 100 nm thick, with a spatial resolution of 40 nm it was proven that curing of the film generated a chemically heterogeneous nanostructure, which is the origin of the nodular structure commonly found in exoxy [75]. Moreover, water uptake was investigated in epoxy-phenolic coatings and nanoscale variations were correlated with the cross-linking density at relative humidities of 35% and 65% RH [76]. The extent of the heat curing was followed by studying the intensity of the peak at 916 cm^−1^, corresponding to the asymmetric oxirane ring deformation mode, and hence a lower intensity corresponds to a higher degree of local curing. The amount of water was estimated by the peak intensity at 3300 cm^−1^, and the by rationing these peaks it was concluded that a high cross-linking density resulted in an enhanced local water uptake. Complementary AFM topography images were obtained simultaneously as the IR spectra, and ATR and DSC were used to further characterize the films. In another study the same research group investigated local water uptake resulting in ion channels in organic films [77]. Such studies are of importance in our understanding of why corrosion inhibiting organic films used for that appear to be unaffected suddenly do not work. By integrating the water band centered at 3416 cm^−1^ in IR reflectance spectra at different times of exposure in 80% RH, it was possible to determine the absorption of water for dry and pre-soaked and then dried films. The presoaked films showed a larger water absorption, a result of the more open structure formed upon presoaking, a structure that was kept when drying. To examine how homogeneous the water uptake was, AFM-IR was used on ~500 nm thick epoxy-phenolic films on a mild steel substrate. A topography map by AFM revealed that immersion in water resulted in a rougher surface and the formation of bumps. When maps of the ratio of the IR signal at 3296 and 3420 cm^−1^ (corresponding to water bands) at 60% and 30% RH were calculated, it was concluded that the bumps corresponded to a higher IR absorbance, and hence a higher local water uptake. The local water uptake in raised polymer regions is also revealed by studying images of the amplitude ratios at the wavenumbers 3420/2964 cm^−1^ and 3296/2964 cm^−1^, where 2964 cm^−1^ is an absorption frequency of the epoxy film, 3420 cm^−1^ corresponds to weakly hydrogen bonded water, and 3296 cm^−1^ originates from strongly hydrogen bonded water. Figure 10 shows an AFM image in (a), which reveals raised regions, amplitude ratios acquired at the wavenumbers 3420/2964 cm^−1^ and 3296/2964 cm^−1^ in (b) and (c), as well as 3296/3420 cm^−1^ in (d).

#### 2.1.7. Photoacoustic Infrared Spectroscopy

Palmer and coworkers developed a setup for photoacoustic IR spectroscopy, which enables in-situ studies in the field and provides information about chemical composition, thickness, quantitative analysis of corrosion products, and layering [78]. Calibration curves for brochantite (Cu_4_SO_4_(OH)_6_) and antlerite (Cu_3_SO_4_(OH)_4_) were obtained based on their infrared absorption frequencies and intensities, and allowed determinations of the brochantite/antlerite ratio with an accuracy of 10%.

### 2.2. Raman Spectroscopy

**Figure 11:**
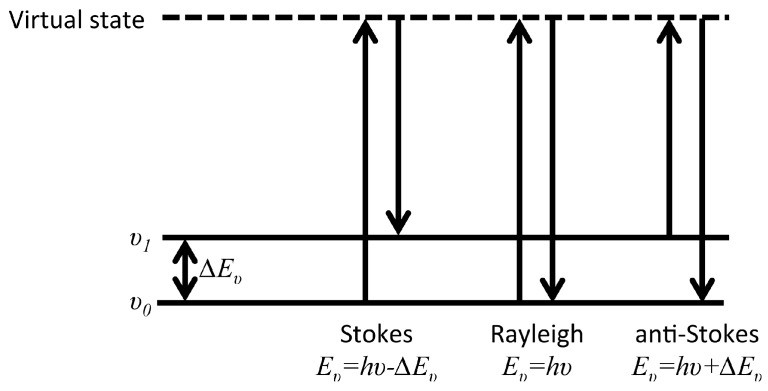
Raman scattering processes. $\nu_{0}$ and $\nu_{1}$ represent the ground and first excited vibrational energy states, respectively, with ∆*E_v_* as the energy difference between them.

After the first experimental observation of Raman scattering by C.V. Raman in 1928 [79], and with the advent of laser, Raman spectroscopy has become a versatile analytical tool in many fields including surface science and corrosion. The Raman process is an inelastic scattering interaction of light with matter and in contrast to infrared processes, Raman occurs off-resonance. Upon excitation of a molecule to an intermediate virtual state, between two stationary exited states, a new photon is scattered from the virtual state accompanied by a relaxation of the molecule. As described in Figure 11, depending on the relative initial and final energy levels, Raman scattering phenomena can be defined as Stokes, Rayleigh, or anti-Stokes. Accordingly, in Raman spectroscopy, the band positions are associated with the difference between the frequencies of the exciting and scattered photon. The selection rule associated with Raman spectroscopy requires a change in the polarizability, α, during a vibration.

#### 2.2.1. Conventional Raman Spectroscopy

##### 2.2.1.1. Steel

Atmospheric corrosion of iron and its alloys including steel has been extensively investigated using Raman spectroscopy. Li et al., characterized the rust formation on 1080 carbon steel after exposure to marine tests with a high concentration of Cl^−^ in Hawaii [80] and utilized micro Raman spectroscopy to identify the main components of the corrosion products, lepidocrocite (γ-FeOOH) in the outer rust layer and goethite (α-FeOOH) and akaganeite (β-FeOOH) in the inner rust layer. Complementary studies using scanning electron microscopy (SEM) and energy dispersive X-ray analyzer (EDXA) on the same point at which Raman spectra were taken enabled them to provide a schematic distribution of rust phases on different samples. They found a significant increase in corrosion rate for deposition rates of Cl^−^ above a certain threshold (75 mg/m^2^/day), which corresponds to the saturation of akaganeite with Cl^−^. Below this threshold the corrosion rate of carbon steel samples was found to be independent of the Cl^−^ deposition rate. The role of critical concentration of Cl^−^ in the formation of akaganeite was also recently observed by Dhaiveegan et al., where the akaganeite corresponding Raman band appeared only after 2 years of exposure of 316 L and 304 stainless steels to industrial-marine-urban environment [81]. It was also showed that the characteristics of the rust layer on mild steel depend on the atmosphere salinity (chlorine ion deposition rate). At low salinity, an adherent rust layer is formed while for high salinity levels, the rust layer can easily exfoliate [82]. Raman peak positions obtained on different corrosion products of rust compounds are tabulated in reference [82]. Li et al., also investigated the very initial stages of NaCl particle induced atmospheric corrosion on 1080 carbon steel [83] combining in-situ and ex-situ Raman spectroscopy with SEM and optical microscopy. They found that the corrosion process starts with localized anodic and cathodic sites where green rust is formed in the regions close to anodic sites, lepidocrocite is mainly formed in the cathodic sites and magnetide (Fe_3_O_4_) is formed at the transition regions between anodes and cathods. The multilayer structure of the corrosion products was also observed on weathering steels with high concentration of copper, chromium, and nickel exposed to marine environments [84]. SEM-EDX analysis confirmed that nickel is distributed throughout the whole corrosion layer while the chromium concentration is higher at the inner part of the corrosion products. The innermost Cr-substitute geolite layer was believed to form the protective rust layer [85,86] limiting the penetration of the corrosive species toward the substrate. Superparamagnetic maghemite was also reported, based on Raman and Mössbauer spectroscopy, to exist in the inner layer of corrosion products and act as a protective layer [87]. Combined with X-ray diffraction (XRD) measurements it was found that lepidocrocite is the main compound of the outer corrosion product layer while the inner part was composed of ferrihydrite/low crystallized magnetite and goethite [88]. Similarly, higher amount of nickel in the composition of the weathering steels results in a greater corrosion resistance in marine environment by increasing the proportion of nanophasic or superparamagnetic goethite in the inner rust layer [89]. Hazan et al., also studied the atmospheric corrosion of AISI-4340 steel upon heat treatment in a high temperature and observed an intermediate layer between the outer wustite and the inner magnetite layers composed of small magnetite islands (bright phase) embedded in a wustite matrix (darker gray) [90].

In the presence of SO_2_ and humidity in the atmosphere, rust layers on iron undergo a phase transition. Such a phase transition was followed using in-situ Raman spectroscopy [91]. It was found for instance that Fe(OH)_3_ which initially is formed in the presence of several sulfur compounds is first transformed to an amorphous FeOOH, which later is crystallized by water loss. Based on these findings a minor modification to Evans model of atmospheric corrosion[92] was proposed.

Aramendia et al., used in-situ an hand held Raman spectrometer to study the rust formation and atmospheric corrosion of sculptures exposed to different conditions in the north of Spain [93]. They found goethite (α-FeO(OH)) as the most stable phase in the corrosion products, accompanied by lepidocrocite, hematite (α-Fe_2_O_3_), and magnetite. For the sculptures exposed to marine sites limonite (FeO(OH)-nH_2_O) and akaganeita (b-FeO(OH)) were also identified. The same group used a dual laser wavelength (785 and 532 nm) portable Raman spectrometer in combination with a hand-held X-ray fluorescence spectrometer and chromatographic analysis to study the corrosion of medieval metallic artifacts from the 13th century [94]. The dual laser wavelength enabled identification of the Cu-based corrosion products phases such as cuprite, malachite, and bronchite (using 532 nm laser) together with Fe based corrosion products such as magnetite, goethite, lepidocrocte, and akaganeite (using 785 nm) on the same probe point of the sample.

Yucel et al., compared the atmospheric corrosion resistance of historic nails made of Ottoman period steels from 16th to 19th centuries and used micro-Raman spectroscopy to identify the composition of the inner and outer layer of the corrosion products [95]. They found that, a compact geolite layer is formed covering most of the inner corrosion layer resulting in enhanced corrosion protection in the Ottaman steel compared with current low alloy steels, indicating the success of iron metallurgy in that time. Although most of the atmospheric corrosion studies using Raman spectroscopy are limited to qualitative analysis, some limited works are available where Raman measurements lead to quantitative parameters. As an example, Monnier et al., analyzed ancient corroded iron samples and established quantitative composition 2D maps by mean of a home-developed software[96] based on spectral decomposition of experimental spectra by a linear combination of reference spectra. Applications of Raman spectroscopy in long term atmospheric corrosion studies on archeological iron samples and analysis of the ancient rust layers are not only important for the purpose of restoration of historical objects, but it also allows deeper understanding of long term corrosion mechanisms under atmospheric conditions. Such an understanding is important in the prediction and modeling of atmospheric corrosion of containers for storage of nuclear waste material [97].

##### 2.2.1.2. Copper

Hayez et al., were the first group to explore the possibilities offered by Raman spectroscopy in studying the atmospheric corrosion of bronze statuary taking into account different types of atmospheric exposure conditions. They set up a Raman spectral database for compounds formed as the result of atmospheric corrosion of copper and its alloys in sulfur containing environments. This database includes different species of copper sulfates such as antlerite, brochantite, posnjakite, langite, and chalcanthite. The identified corrosion products on objects (specimens from sculptures) naturally exposed to an atmospheric environment were comparable to those obtained on copper (II) sulfate minerals [98]. Furthermore Hayez et al., investigated the impact of patination ingredients, the presence of additional elements like iron sulfate or chloride ions as well as the patination method on the chemical composition and nature of artificial patina using non-destructive Raman measurements [99]. The results obtained on artificial patinas were compared to the results obtained on reference products with known composition. Identification of the exact chemical component of artificial patina is a key parameter in restoration purposes, when replacement of a damaged naturally formed patina with an artificial one is required.

Bernardi et al. [100], with an innovative approach, comparatively investigated corrosion of G85 bronze in either acid rain solution collected from natural rain or in synthetic rain containing main organic components (HCOO^−^, CH_3_COO^−^) and the aggressive inorganic components, H^+^, Cl^−^, NO_3_^−^ , NH_4_^+^, SO^2−^_4_ mimicking the natural rain. They pointed out the effect of each alloying element in general corrosion behavior. As a result they found out that there is a slight difference between the corrosion of samples exposed to natural rain compared to those exposed to the synthetic rain. For instance, the identified corrosion products on samples exposed to natural rain were cuprite (Cu_2_O), cerussite (PbCO_3_), litharge (PbO), brochantite (Cu_4_SO_4_(OH)_6_), and devillina (CaCu_4_(SO_4_)^2−^(OH)_6_·3H_2_O), whereas on samples exposed to the synthetic rain cuprite, mixed lead sulphates, and cerussite were identified in the corrosion products.

##### 2.2.1.3. Silver

As a part of a larger project dealing with initial stages of atmospheric corrosion of silver, Martina et al., presented a catalogue of Raman spectra of silver compounds formed during atmospheric corrosion of pure silver using micro-Raman spectroscopy [101]. In this effort, especially for highly photosensitive compounds, micro-Raman spectroscopy with modulated laser intensity is advantageous as a nondestructive analytical tool. In this effort reference Raman spectra for the following silver compounds were obtained: Silver oxide (Ag_2_O), silver (I) chloride (AgCl), silver (I) sulfide (Ag_2_S), silver (I) sulfite (Ag_2_SO_3_), silver (I) sulfate (Ag_2_SO_4_), silver(I) carbonate (Ag_2_CO_3_), silver(I) acetate (AgC_2_H_3_O_2_), and silver(I) nitrate (AgNO_3_). These reference spectra can be used in corrosion studies of both pure silver and silver alloys. Furthermore, with the aim of understanding the role of environmental conditions promoting the atmospheric corrosion, pure silver was exposed to different controlled laboratory atmospheres (synthetic air, different relative humidity (50% and 90%), SO_2_ (500 ppb), and H_2_S (500 ppb)) and formation of corrosion products with the thickness of only several monolayers was followed using micro Raman spectroscopy [102]. After even 24 h exposure of silver to SO_2_ containing humidified air (with 50% RH or 90% RH) silver sulfate and silver sulfite were not identified as proper corrosion products. This reflects the low reactivity of silver towards SO_2_. Comparing Raman spectra obtained on smooth silver exposed to 90% RH and those obtained on scratches on silver exposed to 50% RH revealed a secondary corrosion mechanism involving hydration reaction and chemisorption of gaseous species (oxygen, SO_2_, and CO_2_) in the water allayer. In contrast to these results, when silver was exposed to H_2_S containing humidified air a high reactivity of silver toward tarnishing (i.e., formation of Ag_2_S) was observed, which was enhanced in the presence of higher amounts of humidity. Unlike the SO_2_ exposure case, where the occurrence of secondary atmospheric corrosion processes was detected, after exposure of silver to H_2_S such a process was not observed.

##### 2.2.1.4. Zinc

Ohtsuka et al., [103] investigated the effect of relative humidity (RH) on the formation of corrosion products on zinc in the presence of NaCl precipitations on samples using in-situ Raman spectroscopy. They identified amorphous zinc oxide as the main corrosion product when zinc was exposed to dry air. In a RH around 75%, amorphous ZnO, zinc carbonate (ZnCO_3_)_2_(Zn[OH]_2_)_3_ and traces of Zn(OH)_2_ were identified. However, corrosion of zinc in presence of NaCl and high RH (>80%) was more complicated and occurred in different stages. In the first stage zinc chloride (ZnCl_2_) forms on the surface while zinc hydroxy chloride or simonkollite Zn_5_(OH)_8_Cl_2_·H_2_O was formed as more advanced corrosion products. The formation of simonkollite included dissolution of NaCl particles, electrochemically coupled reactions and precipitation of concentrated zinc chloride.

Jayasree et al., studied the corrosion on zinc sheets exposed in marine exposure sites using FTIR and FT-Raman spectroscopy and identified two compounds, NaZn_4_Cl(OH)_6_SO_4_·6H_2_O and Zn_4_Cl_2_(OH)_4_SO_4_·5H_2_O in the corrosion products [104]. They attributed the observation of multiple bands in the *υ*_s_ SO_4_ vibrational mode region (433, 466, and 497 cm^−1^ and its overtone at 887, 905 and 955 cm^−1^) to a distorted structure for SO_4_ anions in the corrosion products. The amount of distortion was higher in Zn_4_Cl_2_(OH)_4_SO_4_·5H_2_O as a result of stronger hydrogen bonding network in its structure compared to NaZn_4_Cl(OH)_6_SO_4_·6H_2_O.

**Figure 12:**
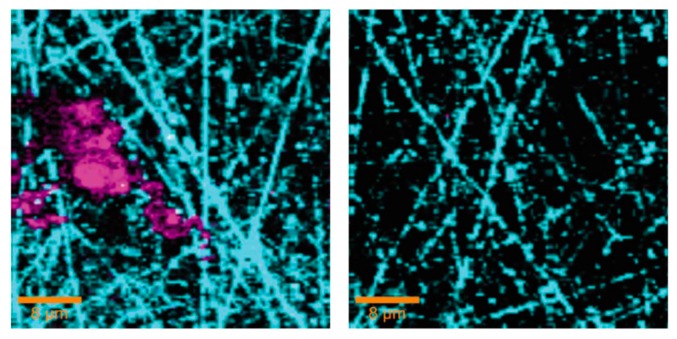
Color-coded Raman images for Zn exposed to 115 ppb acetic acid for 48 h in humid (**left**), and dry conditions (**right**). Purple color represents zinc hydroxy acetate (integrated 2850–3000 cm^−1^) and blue color ZnO (300–650 cm^−1^). The image size is 40 × 40 μm. Reproduced from reference [105] by permission of The Electrochemical Society. Copyright 2010.

Confocal Raman spectroscopy and micro spectroscopy were used to provide a laterally resolved chemical map of the localized corrosion products on zinc exposed to organic volatile solvents (formic acid and acetic acid) in dry and humidified air [105,106]. The identified corrosion products on samples exposed to acetic acid containing humid air included three dimensionally grown zinc hydroxy acetate Zn_5_(OH)_8_(CH_3_COO)_2_·4H_2_O and zinc oxide distributed heterogeneously on the surface, as shown in Figure 12. Similarly, on the samples exposed to humidified air and formic acid, zinc oxide and zinc hydroxy formate were identified. However, on the sample exposed to dry air containing either acetic acid or formic acid, only zinc oxide was identified in the corrosion products. The distribution of zinc oxide was found to be more uniform compared to zinc hydroxy carboxylate.

**Figure 13:**
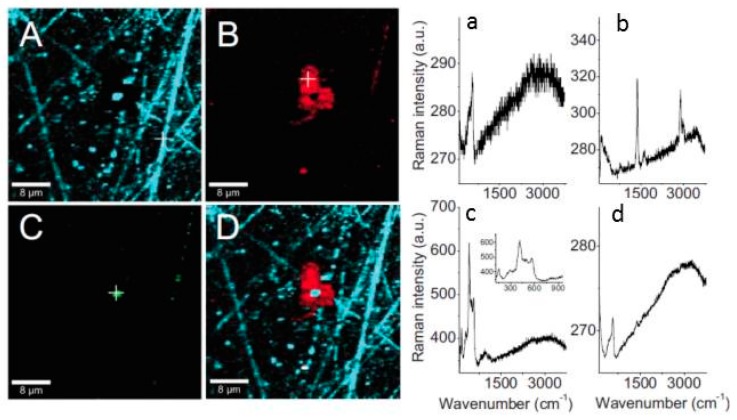
Left: Color-coded Raman images for zinc exposed to 100 ppb formic acid in humid air (90% RH) for 2 h. (**A**) ZnO (300–620 cm^−1^); (**B**) CH in Zn hydroxy formate (2850–3000 cm^−1^); (**C**) The region 380–480 cm^−1^; (**D**) The combination of the Raman images in **A**–**C**. Right panel (**a**–**c**): Raman spectra corresponding to the marked spots in the color-coded images; (**d**) is the average over the whole image. Image size 40 × 40 µm. The Raman images were collected ex situ. Reproduced from reference [106] by permission of The electrochemical Society. Copyright 2010.

**Figure 14:**
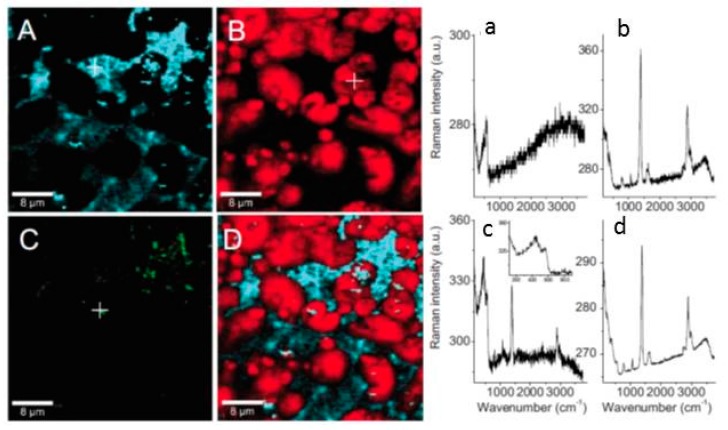
Left: Color-coded Raman images for zinc exposed to 100 ppb formic acid in humid air (90% RH) for 2 h (upper part) and 48 h (lower part). (**A**): ZnO (integrated 300–620 cm^−1^); (**B**): CH in Zn hydroxy formate (2850–3000 cm^−1^); (**C**): more crystalline ZnO (380–480 cm^−1^); (**D**): the combination of the Raman images in **A**–**C**. Right panel (**a**–**c**): Raman spectra corresponding to the marked spots in the color-coded images. (**d**) is the average over the whole image. The image size is 40 × 40 microns. Reproduced from reference [106] by permission of The electrochemical Society. Copyright 2010.

An interesting observation from the confocal Raman microspectroscopy results obtained on zinc samples exposed to humidified air containing formic acid for 2 h is the different distribution of crystalline and amorphous zinc oxide as well as the zinc hydroxy formate (Figure 13). It was evident that more crystalline zinc oxide forms in the center of zinc hydroxy formate, while the amorphous zinc oxide is almost homogeneously distributed on the surface. After prolonged exposure (up to 48 h), the average Raman spectrum is dominated by copper hydroxy formate and Raman microspectroscopy shows that the zinc oxide and zinc hydroxy formate are still clearly separated, as seen in Figure 14.

**Figure 15:**
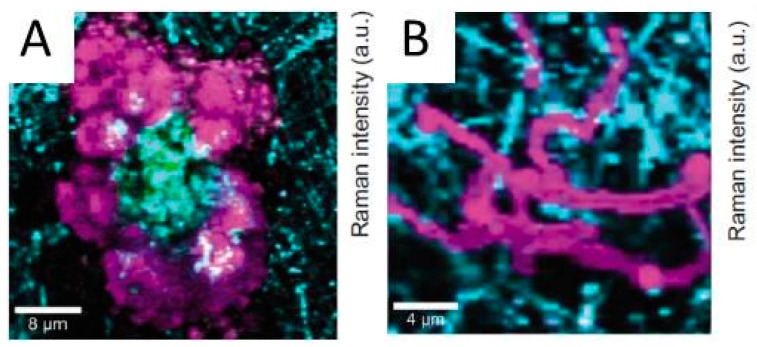
Formation of ring like (**A**) and filament-like corrosion products, characteristic of filiform corrosion (**B**). Reproduced from reference [106] by permission of The electrochemical Society. Copyright 2010.

When zinc samples were exposed to humidified air containing acetic acid for the period of 2 h, similar to the case of formic acid exposure, zinc oxide and zinc acetate form as corrosion products. However, unlike the formic acid exposure case where corrosion products were homogenously distributed on the surface, upon prolonged exposure (48 h) of zinc to acetic acid containing humidified air corrosion products form in two distinct morphologies: ring like disks and filaments, as shown in Figure 15. The ratio between different bands in the Raman spectra of these two aggregates indicates that zinc hydroxy acetate is formed with slightly different structures.

##### 2.2.1.5. Zinc Alloys

Atmospheric corrosion of duplex brass (Cu-20Zn) upon exposure to humidified air (90% RH) was studied by confocal Raman micro spectroscopy, IRRAS, scanning Kelvin probe force microscopy (SKPFM), atomic force microscopy (AFM), and scanning electron microscopy with energy dispersive X-ray analysis (SEM/EDS) [60]. Confocal Raman micro spectroscopy results demonstrated that after 3 days of brass exposure to humidified air, amorphous zinc oxide protrudes from more uniform copper (I) oxide (Cu_2_O) on the surface. This distribution of oxides at the surface was attributed to variations in nobility along zinc rich and copper rich areas which result in galvanic effects. As a result, local growth of zinc oxide is accelerated and retards a more uniform growth of copper (I) oxide.

**Figure 16:**
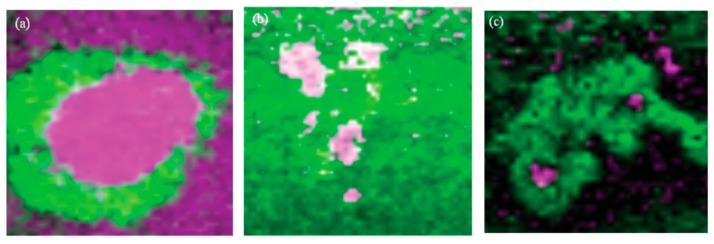
(Color online) Raman images of brass exposed 1 month to humidified air with formic acid (**a**); acetic acid (**b**); or propionic acid (**c**). The formation of Zn-carboxylate is represented by pink and of Cu_2_O by green. Scan size 10 × 10 µm. Reproduced from reference [61] by permission of The electrochemical Society. Copyright 2010.

As an extension of the previous study, duplex brass (Cu-20Zn) samples were exposed to humidified air containing carboxylic acids (formic, acetic, and propionic acid) [61]. Raman micro spectroscopy together with IRRAS investigations revealed that the main corrosion products include copper oxide and zinc carboxylates (e.g., Zn(HCOO)_2_·*x*H_2_O or Zn_5_(OH)_8_(CH_3_COO)_2_·*x*H_2_O or Zn-propionate). Investigations of the lateral distribution of the corrosion products on the samples after 3 days exposure to humidified air containing carboxylic acids indicated that the corrosion products are formed in a cell like structure, where centrally located zinc carboxylates are in general surrounded by areas of copper (I) oxide. The size of the identified corrosion cells on brass samples were in the order formic acid > acetic acid > propionic acid, which reflects the importance of ionic conductivity of the aqueous allayer (Figure 16).

**Figure 17:**
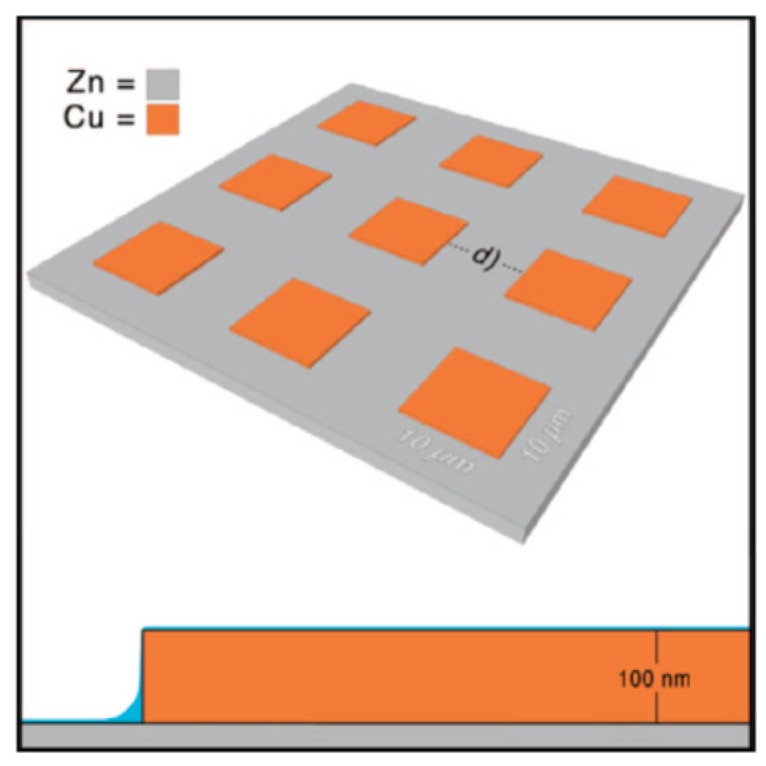
A schematic illustration of copper islands (brown) on zinc substrate (gray). Reproduced from reference [107] by permission of The Electrochemical Society. Copyright 2013.

**Figure 18:**
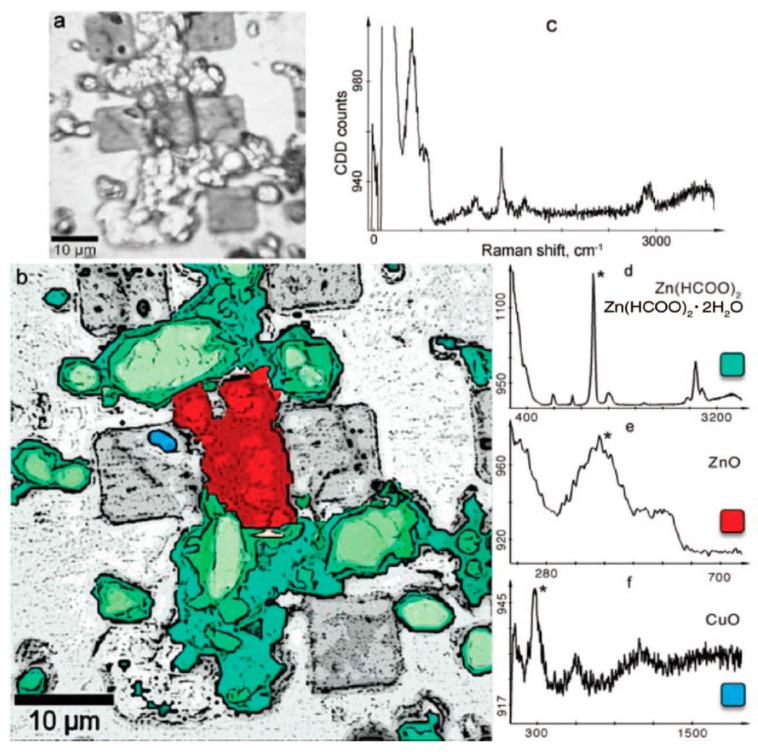
Confocal Raman image analysis of a patterned sample exposed for 5 days in 100 ppb formic acid in 80% RH. (**a**) An optical microscope image of the analyzed area; (**b**) A summary image of the local distribution of species based on interpolation of their characteristic Raman signals. The green color corresponds to interpolation between 1325 and 1435 cm^−^^1^ (**d** *) and denotes Zn(HCOO)_2_ and Zn(HCOO)_2_·2H_2_O. The red color corresponds to interpolation between 320 and 490 cm^−^^1^ (**e** *) and denotes poorly crystalline ZnO. The blue color corresponds to the interpolation between 250 and 320 cm^−1^ (**f** *) and denotes CuO; (**c**) An average spectrum of the red area. On the right-hand side there are average spectra (**d**–**f**) for the presented species in (**b**). Reproduced from reference [107] by permission of The Electrochemical Society. Copyright 2013.

Because of the increased need to understand the micro-galvanic effects during atmospheric corrosion of alloys, well defined patterned copper-zinc samples (25Cu-74Zn) were studied as a simple model for brass, as shown in Figure 17 [107]. After exposure of such patterned sample to humidified air (80% RH) containing formic acid for 5 days, the distribution of the corrosion products were evaluated by mean of confocal Raman microspectroscopy, displayed in Figure 18. The identified corrosion products included crystalline zinc oxide, zinc formate Zn(HCOO)_2_, and hydrated zinc formate Zn(HCOO)_2_·2H_2_O, as well as a very small amount of copper (II) oxide. In the corrosion products no copper (I) oxide was detected since its amount was below the detection limit. Based on the Raman peak position for the out of plane CH bending mode, which enables distinguishing between zinc formate and hydrated zinc formate [61], it was concluded that both compounds exist in the corrosion products. The combined microscopic and spectroscopic techniques in this study provided detailed information regarding the micro-galvanic coupling effect in atmospheric corrosion of brass. A clear separation between zinc oxide and zinc formate products was observed in the zinc region between copper islands where the local chemistry governs nucleation and growth of corrosion products and surface energy favors the formation of radially grown corrosion products.

When a similarly patterned sample was covered with a self-assembled monolayer of octadecanethiol (ODT) as a corrosion inhibitor before exposure to humidified air containing formic acid, the formation rate of corrosion products was initially decreased [108]. However, prolonged exposure resulted in disordering/removal of the ODT layer and an accelerated corrosion of the sample, as shown by VSFS. After 5 days of exposure Raman microspectroscopy showed a clear different distribution of corrosion products depending on the distance from the copper/zinc interface. In areas adjacent to the copper islands, hydrated zinc formate (Zn(HCOO)_2_·*x*H_2_O) is predominant, while hydrated zinc hydroxy formate (Zn_5_(OH)_8_(HCOO)_2_·*x*H_2_O) as well as great amounts of adsorbed water were identified in the more extended corrosion products further away from copper islands. The distribution of zinc formate over the whole distance range from copper islands was found to be more or less uniform. Such a distribution of corrosion products can be directly connected to the micro-galvanic effect as well as ion migrations during corrosion.

##### 2.2.1.6. Other Elements and Alloys (Mo, Ni, Pb, SiC Fibers, U, Mg, Al, CdS, Graphene, As_2_S_3_)

Atmospheric corrosion of other metals than abovementioned alloys and metals have been also studied by Raman spectroscopy to a lesser extent and only very few examples of such studies exist in the literature on Mo, Ni, Pb, U, Mg, and Al.

The effect of formaldehyde in the indoor atmospheric corrosion of lead was studied by de Faria et al., [109]. Using a combination of SEM and Raman spectroscopy they showed that formats are produced as the results of exposure of lead coupons to formaldehyde and that oxidants such as H_2_O_2_ are not necessary. They observed significantly more complex Raman spectra for the corrosion products compared to the simple Pb(HCO_2_)_2_ spectrum.

#### 2.2.2. Surface Enhanced Raman Spectroscopy (SERS) and Tip Enhanced Raman Spectroscopy (TERS)

Raman spectroscopy, despite its applications in determining the structure and composition of corrosion products suffers from a major challenge which is the very small signal to noise ratio if the number of scatterers are limited. This can be problematic especially when the intention is investigation of very thin corrosion products. However, enhancing the electric field either by roughening the sample surface (surface enhance Raman spectroscopy or SERS) or using a sharp tip as an antenna (tip enhanced Raman spectroscopy or TERS) allows huge enhancement of the Raman (by a factor of 10^6^) so that even single molecule detection becomes possible. Although there have been some efforts in using SERS and TERS in a combination with electrochemistry, very limited studies exist where enhanced Raman spectroscopy has been applied to study the atmospheric corrosion of metals. For instance, it has been shown that the atmospheric oxidation of silver, as the SERS substrate, in an ambient laboratory exposure air affects the enhancement factor dramatically such that the SERS signal intensity drops by more than 60% after only five hours exposure [110]. However, the main emphasis of this work is not studying of the corrosion of silver, but rather to emphasize the importance of substrate oxidation and contamination. Similarly, although silver is considered as a very good candidate to act as an antenna for enhancement of the Raman signal, its oxidation when used as the tip in TERS measurements in air, causes dramatic plasmonic degradation [111]. Therefore the same tip cannot be used for long term measurements and also cannot be stored.

One of the very few examples where SERS has been actually used in studying surface species upon corrosion is the work by Wang et al., [112] where a combination of in-situ, ex-situ SERS, and cathodic stripping voltammetry was used to study the effect of different exposure media including aerated 0.15 M NaCl, 0.10 M NaOH solutions, and air on the formation of corrosion product on molybdenum as well as Mo dissolution rate. The surface species were identified as molybdenum (IV) oxide, molybdate, and heptamolybdate. They also found that Mo, especially in basic solutions is not well passivated. Furthermore, Tormoen et al., [113] examined real time adsorption of volatile corrosion inhibitors on different substrates using in-situ SERS and concluded that below a relative humidity level of about 20% no adsorption of volatile corrosion inhibitors occurred regardless of the substrate.

### 2.3. Vibrational Sum Frequency Spectroscopy (VSFS)

A great challenge in studying surface reactions including oxidation and corrosion is that in most of available spectroscopic methods the signal generated from interfacial molecules is overwhelmed by the signal arising from the vast number of bulk molecules. Advances of non-linear spectroscopic tools including vibrational sum frequency spectroscopy (VSFS) and second-harmonic generation (SHG) allows probing surfaces and interfaces with no bulk signal contribution [114].

**Figure 19:**
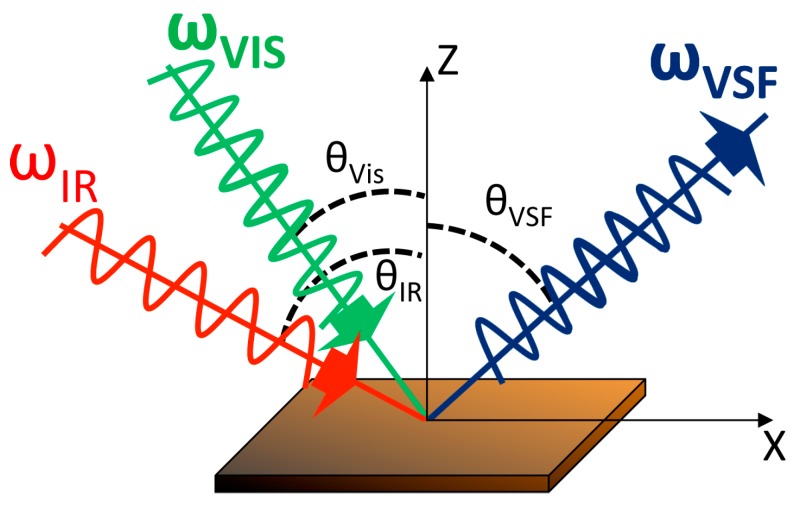
Schematic of the vibrational sum frequency spectroscopy (VSFS) geometry. The θ values represent the incident or reflected angles from the surface normal for visible, IR, and VSF beams. Each of the beams can be polarized perpendicular to the plane of incident (S-polarized) or parallel to it (P-polarized).

Briefly, as shown in Figure 19, in VSFS two laser beams (usually visible and IR) are spatially and temporarily overlapped on the sample surface and a third beam with its frequency equivalent to the frequency of the incoming beams is generated (ω_VSF_ = ω_Vis_ + ω_IR_). Under the dipole approximation, the sum frequency generation is not allowed in the centrosymmetric media including bulk materials where dipoles are randomly oriented. However, at surfaces and interfaces, due to the broken symmetry, a VSF signal is generated making this process inherently surface sensitive. Furthermore, so called non-resonant response in the VSF signal and its interference with the resonant signal can be used to probe the oxidation of the surface [115]. Besides, VSF measurements using a combination of S and P polarized beams allow an orientation analysis of the adsorbates on a surface to be performed. Therefore, VSFS is a suitable technique in studying corrosion and oxidation on a molecular level. In this respect, few studies have been specifically devoted to studying the atmospheric corrosion of metals.

#### Atmospheric Corrosion Studies by VSFS

**Figure 20:**
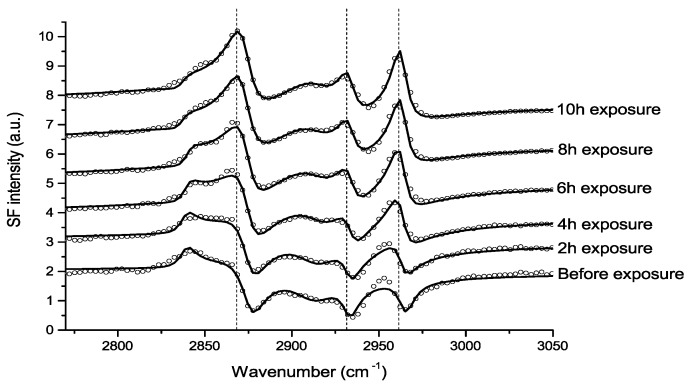
Time evolution of VSF spectra of the octadecanethiol (ODT) covered copper during exposure to dry air. Experimental data and fitted spectra are presented as empty circles and lines, respectively. The CH_3_ symmetric, Fermi resonance, and antisymmetric stretches from ODT molecules are marked with the dash lines. The spectra are offset for clarity. Reprinted with permission from reference [115], Copyright 2011, American Chemical Society.

Hosseinpour et al., in series of studies investigated the initial stages of atmospheric corrosion of copper coated with a self-assembled monolayers (SAMs) as corrosion inhibitors, using a combination of IRAS, Raman, VSFS, quartz crystal microbalance (QCM), indirect nanoplasmonic sensing (INPS), and cathodic reduction. They adsorbed a SAM of octadecanethiol (ODT) on nonoxidized copper and observed a dramatic change in the appearance of the VSF spectra upon exposure of the sample to dry air. Qualitatively they attributed these changes to the formation of a very thin layer of copper (I) oxide underneath the ODT layer. Reducing the formed oxide in an electrochemical cell and comparing the results with in-situ IRRAS measurements they quantified less than a nanometer as the upper limit for the thickness of the oxide layer [115]. In Figure 20 the gradual change in the shape of VSF spectra upon oxidation of ODT coated copper during exposure to dry air is depicted. This provides an indirect study of the formation of Cu_2_O, where the spectral changes are due to the fact that Cu and Cu_2_O have a different non-resonant signal which thus interferes differently with the CH_2_ and CH_3_ signal from the ODT chains.

**Figure 21:**
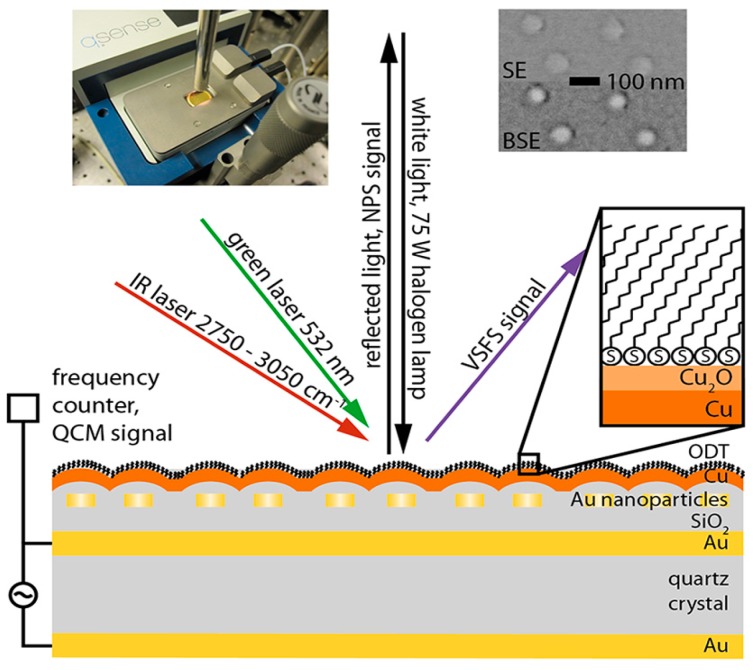
A schematic of the combined QCM-D and indirect nano plasmonic sensor, serving also as the VSFS sample. On the upper electrode of the QCM sensor, the Au nanoparticles for INPS are embedded in a SiO_2_ film. On top of this film the actual sample is deposited, a Cu film with a corrosion protective ODT layer. The magnified picture of the latter shows the surface of the Cu film with its layer of Cu_2_O and with the ODT layer on top. The measurement setup is shown on the upper left including an image of the QCM-D window module with the copper coated quartz crystal-Au-nanoparticle-sensor and the reflection probe for INPS. The two SEM images (upper right), one taken with a secondary electron (SE) detector and the other with a backscattered electron (BSE) detector, show the sample surface and the INPS sensing Au nanoparticles. The low contrast in SE points to the fact that the Au nanoparticles are well covered by the SiO_2_ layer and have no major influence on the surface roughness. Reprinted with permission from reference [117], Copyright 2013, American Chemical Society.

Integration of VSFS with a mass sensitive technique (QCM) allowed an in-situ quantification of the oxide layer thickness [116]. Hosseinpour et al., further concluded that the initial oxidation of ODT covered copper under dry air atmosphere occurs in two steps. The initial fast oxidation step was attributed to the penetration of oxygen through the ODT layer from easily accessible pathways, probably related to the gauche defects in the SAM layer. This initial fast step was followed by a slower oxidation rate with the rate limiting factor being the oxygen penetration through the SAM layer. Overall the VSFS-QCM combination allowed a quantification of oxide layer formation on copper with the resolution of 5% and 2% of an ideal cuprite layer for VSFS and QCM, respectively. Schwind et al., used an in-situ combination of VSFS-QCM and INPS to study the atmospheric corrosion and oxidation of ODT covered copper in both dry air and humidified air [117]. They found that introduction of humidity increases the oxidation rate by a factor of 4–5. Based on the VSFS results no significant change in the ordering and configuration of the protective SAM layer was observed in the course of copper oxidation. In contrast, when copper covered with alkanethiol SAMs of different chain lengths were exposed to a more corrosive atmosphere, consisting of 100 ppb formic acid and air with 85% relative humidity, an increased disordering in the SAM structure was observed during the corrosion process [118]. In Figure 21 the integration scheme for VSFS-QCM and INPS is depicted. This figure shows the advantage of integration of multiple techniques for in-situ measurements. An enhanced corrosion protection efficiency was observed for SAMs with longer chain lengths. It was also observed that the deposition of SAMs on the copper surface within experimental error completely stops the formation of copper (I) oxide, which is in stark contrast in comparison with bare copper. Such a retardation was less efficient against the formation of copper formate and copper hydroxide. VSFS studies showed that unlike alkanethiols which could be deposited on copper surfaces without any copper oxide being formed, SAMs of alkaneselenols (selenol is located below sulfur in the periodic system) always co-existed with copper (I) oxide at the surface [119]. This was attributed to the less efficient ability of alkaneselenols in replacing the very thin initial oxide on the copper surface during their adsorption. Nevertheless, both alkanethiols and alkanselenols initially formed order structures on the copper/copper oxide surface, as revealed by VSFS. However, upon prolonged exposure of the SAM covered samples to humidified air containing 100 ppb formic acid, the alkaneselenols were partially removed from the copper surface, while alkanethiols, though less ordered, remained on the surface as a protective layer. Consequently, no copper (I) oxide was observed on alkanethiol covered copper while a substantial amount of copper (I) oxide was identified on alkaneselenol covered copper after partial removal of the protective molecules from the surface due to the formation of localized galvanic couples (i.e., areas with and without alkaneselenols). VSFS studies on brass (Cu_20_Zn and Cu_40_Zn) also showed that ODT can form ordered protective monolayer on both single and double phase Cu-Zn alloys [120]. The conformation of the protective layer on these samples did not change dramatically upon the sample exposure to humidified air containing formic acid. It was also showed that local galvanic effects on double phase brass resulted in less efficient corrosion inhibition [120]. However, due to the lack of lateral resolution in these VSFS measurements a direct comparison between the local structure of SAM and the induced inhibition was not feasible. Santos et al., utilized the lateral resolution offered by VSFS imaging microscopy to analyze the distribution of the copper oxide growth underneath a SAM of ODT upon spontaneous atmospheric oxidation [121]. Their findings also indicated that as the result of copper oxidation, the overall mean tilt angle of the ODT molecules at the surface decreases and the amount of gauche defects in the SAM structure increase. Furthermore, they observed a heterogeneous distribution of ODT molecules with different degrees of ordering, suggesting that oxidation of ODT covered copper is locally heterogeneous initiated from the domain boundaries moving inward.

Hedberg et al., also used in-situ VSFS combined with IR studies and density functional theory (DFT) calculations to understand the initial stages of atmospheric corrosion of zinc initiated by formic acid in dry as well as humid conditions [122]. They found that adsorption of formic acid onto Zn/ZnO surface occurs independent of the presence or absence of humidity through a ligand exchange process. However, the presence of humidity accelerated the growth of the corrosion products layer, including formate. Their DFT calculations supported the idea of coordination of the formate to zinc ions without the participation of water molecules. They also found that formic acid is partly dissociated to fomate ions during adsorption to a ZnO surface with different hydration states depending on the relative humidity. The orientation analysis of zinc formate revealed that in humid conditions formate is ordered with the oxygen atoms toward the ZnO surface and the C-H moiety away from the surface [123]. Furthermore, VSFS studies provided evidence of zinc hydroxylation and gradual replacement of the hydroxyl groups by formate species, representing the initial stages of zinc dissolution induced by formic acid [124].

Very recently Gretic et al., applied self-assembled monolayer and multilayers of stearic acid as a corrosion protection system for copper-nickel alloy in a simulated marine exposure condition. They combined the results from electrochemical studies, AFM measurements, and contact angle measurements with those obtained from ellipsometry to assess the efficiency of the protective layers and their average thickness, respectively. In their studies, VSFS measurements were performed to assess the degree of ordering of the deposited layers on the copper-nickel surface using non-resonant suppression method developed by Lagutchev et al.[125] They found that the efficiency of the mono-/multi- layers in protecting the alloy surface from corrosion was highly dependent on the number of the layers as well as their homogeneity [126].

## 3. Conclusions

In this review article, we have summarized the main part of the work done during the last decades where vibrational spectroscopy has been employed to investigate atmospheric corrosion phenomena. A wide range of samples and exposure conditions, from outdoor atmospheric corrosion studies on exposed sculptures and historical objects to indoor and controlled exposure in-situ studies have been covered. In many cases, reference compounds are synthesized in laboratories and their spectra are compared with the sample spectra to characterize the corrosion products formed on objects with more extended and complicated corrosion. In addition, other kinds of techniques able to provide complementary information, such as the morphology or the mass of the corrosion products have been mentioned. Most of the studies presented here rely on vibrational spectroscopic techniques with a low spatial resolution (mm–cm), and hence the information obtained is an average over a large area. However, in order to further our knowledge in the field, it is desirable that a corrosion process can be studied and chemical information can be obtained at the nano level. This can now be achieved by novel IR and Raman spectroscopy techniques, with a spatial resolution as low as 10–20 nm, and has the possibility to open up new doors in the field of atmospheric corrosion.

## Figures and Tables

**Table 1:** Important corrosive gases in indoor environments [1,2]. Reproduced from [1] by permission of The Electrochemical Society.

Species	Typical indoor Concentration (ppb)	*H* ^b^	Equilibrium Solution Concentration (µM)
O_2_	2.1 (8) ^a^	1.7 (−3)	3.6 (2)
O_3_	18	1.8 (−2)	3.2 (−4)
H_2_O_2_	5	2.4 (5)	1.2 (3)
H_2_S	0.3	1.5 (−1)	4.5 (−5)
COS	0.6	3.7 (−2)	2.2 (−5)
SO_2_	30	1.4	4.2 (−2)
HCl	0.4	2.0 (1)	8.0 (−3)
Cl_2_	0	6.2 (−2)	0
NH_3_	10	1.0 (1)	1.0 (−1)
NO_2_	4	7.0 (−3)	2.8 (−5)
HNO_3_	3	9.1 (4)	2.7 (2)
CO_2_	6.0 (5)	3.4 (−2)	2.0 (1)
HCHO	10	1.4 (4)	1.4 (2)
HCOOH	20	3.7 (3)	7.4 (1)
CH_3_COOH	20	8.8 (3)	8.8 (1)

^a^ 2.1 (8) means 2.1 × 10^8^; ^b^ Henry’s law constant.
